# Secondary Bile Acids Modified by *Odoribacter Splanchnicus* Alleviate Colitis by Suppressing Neutrophil Extracellular Trap Formation

**DOI:** 10.1002/advs.202509073

**Published:** 2025-09-24

**Authors:** Jing Xu, Jianhong Li, Xue Guo, Chen Huang, Yao Peng, Haoming Xu, Yingfei Li, Jingkui Xu, Jinxia Hu, Yitong Liao, Yuqiang Nie, Youlian Zhou

**Affiliations:** ^1^ Department of Gastroenterology and Hepatology the Second Affiliated Hospital School of Medicine South China University of Technology Guangzhou Guangdong China; ^2^ Department of Gastroenterology and Hepatology Guangzhou First People's Hospital South China University of Technology Guangzhou Guangdong China; ^3^ Department of Gastroenterology and Hepatology Shenzhen University General Hospital Shenzhen University Xueyuan AVE 1298, Nanshan District Shenzhen 518055 China

**Keywords:** gut microbiota, neutrophil extracellular traps, odoribacter splanchnicus, secondary bile acids, ulcerative colitis

## Abstract

The gut microbiota contributes to inflammatory bowel disease (IBD) pathogenesis, yet the functional impact of specific bacterial species remains unclear. Here, *Odoribacter splanchnicus* (*O. splanchnicus*) is indentified as a taxon depleted in human IBD cohorts and demonstrated its protective effects in acute and chronic murine colitis models. In mice, *O. splanchnicus* administration alleviated colonic inflammation and preserved barrier integrity, accompanied by a restructured mucosal immune landscape and reduced neutrophil extracellular traps (NETs) formation. This inhibitory effect on NETs is lost in *Pad4^‐/‐^* mice, highlighting its dependence on NETs formation machinery. Metabolomic profiling showed that *O. splanchnicus* treatment elevated the secondary bile acid lithocholic acid (LCA). This increase is lost following antibiotic cocktail treatment and restored by fecal microbiota transplantation from *O. splanchnicus*‐treated donors, demonstrating a requirement for an intact gut microbiota. Mechanistically, LCA supplementation recapitulated the anti‐NETs formation phenotype and suppressed colonic inflamation by inhibiting the NLRP3‐GSDMD signaling pathway. Together, these findings define a gut microbiota‐metabolite‐neutrophil axis in IBD pathogenesis, highlighting the microbiota‐dependent regulation of LCA as a key protective mechanism of *O. splanchnicus*.

## Graphical abstract

In ulcerative colitis, the loss of *Odoribacter splanchnicus* disrupts a microbiota‐metabolite‐neutrophil axis. Restoring this bacterium boosts lithocholic acid, which suppress neutrophil extracellular trap formation and eases colonic inflammation. These effects vanish without intact microbiota, underscoring bile acid‐driven NET suppression as a therapeutic strategy.

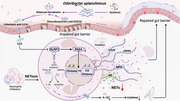

## 1 Introduction

Ulcerative colitis (UC) and Crohn's disease (CD) are the two major subtypes of inflammatory bowel disease (IBD), a chronic, recurrent inflammatory disease characterized by persistent abdominal pain and diarrhea. Currently, there remains a large proportion of patients with IBD who exhibit a poor response to available therapy, including 5‐aminosalicylic acid (5‐ASA), corticosteroids, and biologics or require surgical intervention, placing a significant burden on both patients and society. Hence, it is crucial to investigate the underlying causes of treatment failure in patients receiving basic therapy, as well as the mechanisms driving disease pathogenesis, which might provide strategies to alleviate suffering in patients with UC.

The continuous stimulation of genetically susceptible individuals by environmental factors leads to intestinal barrier damage and chronic inflammation in the host. Accumulating evidence suggests that dysbiosis plays a role in the pathogenesis and development of IBD. For example, *Faecalibacterium prausnitzii*, a short‐chain fatty acid (SCFA)‐producing beneficial bacterium, is depleted in the feces and mucosal tissue of patients with IBD.^[^ ^1^ ^]^ Some studies have found that *Bifidobacterium* alleviates inflammation by regulating immune cells, such as activating dendritic cells (DCs)^[^ ^2^ ^]^ and increasing Tregs.^[^ ^3^ ^]^ Moreover, our research indicated that *Clostridium butyricum* could modulate gut microbiota composition by upregulating beneficial bacteria and downregulating pathogenic bacteria.^[^ ^4^ ^]^ Another IBD‐associated potentially beneficial bacterium is *Akkermansia muciniphila*,^[^ ^5^ ^]^ which plays a role in regulating host barrier function and immune responses. The crosstalk between gut microbiota and the host in IBD primarily relies on microbial metabolism,^[^ ^6^ ^]^ which can be derived directly from bacteria or transformed by gut bacteria to maintain mucosal immune homeostasis and promote gut barrier integrity. However, the protective functions of commensal bacteria in IBD, especially in gut barrier maintenance and immune modulation, remain largely unknown.

Large amounts of antigens entering the intestinal mucosa can trigger excessive immune responses, leading to uncontrolled damage to the host.^[^ ^7^ ^]^ Among the immune cells involved, neutrophils are the first to be recruited to the site of inflammation. By recognizing and eliminating pathogens, as well as releasing neutrophil extracellular traps (NETs), they play a key role in balancing inflammation and mucosal healing.^[^ ^8^ ^]^ In particular, protein arginine deiminase‐4 (PAD4) is activated by various antigens, leading to histone citrullination and subsequent chromatin decondensation and release.^[^ ^9^ ^]^ NETosis, characterized by the release of web‐like structures composed of decondensed DNA and antimicrobial proteins including myeloperoxidase (MPO) and neutrophil elastase (NE), occurs in many medical conditions.^[^ ^10^ ^]^ Nevertheless, excessive NET formation has been observed in the inflamed colons and circulating neutrophils of patients with UC, where it contributes to the persistence of inflammation.^[^ ^11^ ^]^ Some evidence suggested that NETs can impair skin healing in patients with diabetes.^[^ ^12^ ^]^ Furthermore, recent studies indicated that gut microbiota‐derived metabolites, such as indoles, can play a protective role against tissue damage by regulating MPO activity in neutrophils.^[^ ^13^ ^]^ This suggests that specific bacteria and their metabolites may be associated with neutrophil function. However, the specific host‐derived factors that trigger NET formation, as well as the mechanisms underlying gut microbiota‐neutrophil crosstalk in maintaining the mucosal barrier and immune homeostasis, remain to be elucidated.

*Odoribacter splanchnicus* is a member of the phylum *Bacteroidota*. Decreased abundance of *O. splanchnicus* has been associated with non‐alcoholic fatty liver disease (NAFLD), cystic fibrosis, and IBD.^[^ ^14^ ^]^ A recent study showed that *O. splanchnicus* plays a key role in the effectiveness of fecal microbiota transplantation (FMT) in patients with UC.^[^ ^15^ ^]^ By classifying and sequencing IgA‐coated bacteria (IgA‐seq) in fecal samples from FMT donors and recipients, researchers have found that *O. splanchnicus* inhibits colitis in mice by producing SCFAs^[^ ^15^ ^]^ and promoting the differentiation of Tregs.^[^ ^15^ ^]^ Another study identified *O. splanchnicus* as a potential probiotic through metagenome sequencing and verified its protective role in inducing Th17 cells, which are effective against intestinal inflammation and colon cancer.^[^ ^16^ ^]^ However, little is known about whether decreased *O. splanchnicus* is associated with the development of IBD, as well as the potential effects of *O. splanchnicus* supplementation on mucosal healing and immune modulation in IBD.

In this study, we evaluated the clinical significance of *O. splanchnicus* in patients diagnosed with UC. Furthermore, we investigated the functional role of *O. splanchnicus* in inflammation suppression and gut barrier protection using dextran sulfate sodium (DSS)‐induced acute colitis and *Il‐10^−/−^* chronic colitis mouse models. Our findings supported the hypothesis that commensal bacteria regulated inflammatory homeostasis and disease outcomes by modulating innate immunity. Therefore, we investigated whether *O. splanchnicus* influences the immune landscape, particularly in neutrophils, to determine the outcome of inflammation and intestinal barrier integrity. Here, we demonstrated that *O. splanchnicus* modulated colonic immunity by regulating the formation and degradation of NETs, thereby alleviating colitis and mitigating gut barrier injury. Mechanistically, we proposed that *O. splanchnicus* contributed to bile acid metabolism and maintained NET homeostasis through its role in the transformation of the secondary bile acid lithocholic acid (LCA).

## 2 Results

### 2.1 *O. splanchnicus* is Depleted in Patients with UC and is Associated With Specific Clinical Characteristics

**Figure 1:**
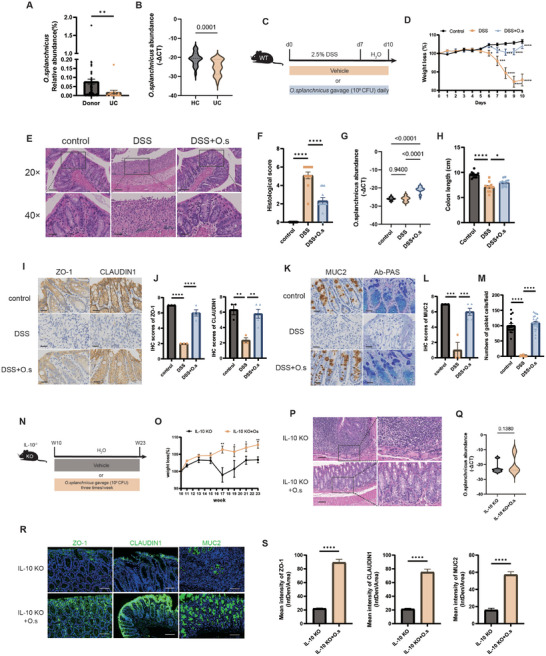
*O. splanchnicus* alleviates inflammation and gut barrier injury in DSS‐induced colitis and *Il‐10^−/−^* mice. A) Comparison of the levels of *O. splanchnicus* in fecal samples from patients with newly diagnosed UC and healthy controls. Data are presented as mean ± SEM. ^**^, *P* < 0.01, as determined by the Mann–Whitney unpaired nonparametric test. B) Violin plot showing a depletion of *O. splanchnicus* in patients with UC in Cohort 2, providing validation of the findings. Data are presented as mean ± SEM. ^****^, *P* < 0.0001, as determined by the Mann–Whitney unpaired nonparametric test. C) Experimental scheme for the intervention of *O. splanchnicus* in the 2.5% DSS‐induced colitis mouse model. Each mice was gavaged with *O. splanchnicus* at a concentration of 10^8^ CFU/200 µL daily for 10 days. *O. splanchnicus* was suspended in sterile PBS, and an equivalent volume of sterile PBS was used as the vehicle control. After the intervention with *O. splanchnicus*, weight loss and DAI scores were recorded and assessed. The distal colon was collected for HE staining, and the remaining colon was reserved for further validation. n = 12 individuals/group. D) Weight loss, E) representative HE staining images at 20× and 40× magnification, F) Comparison of statistical histological scores, G) colonization of *O. splanchnicus* assessed by qPCR, H) statistical analysis of colon length across groups. Data are shown as mean ± SEM. ^****^, *P* < 0.0001, determined by the nonparametric Kruskal–Wallis test. I) Representative IHC staining images for tight junction proteins ZO‐1 and CLAUDIN1, and J) statistical analysis of IHC scores. K) Representative IHC staining images for mucus and goblet cells using MUC2 and AB‐PAS staining, and L,M) statistical analysis of these results. Data are presented as mean ± SEM. ^**^, *P* < 0.01; ^***^, *P* < 0.001; ^****^, *P* < 0.0001, as determined by the nonparametric Kruskal–Wallis test. N) Experimental scheme for the intervention of *O. splanchnicus* in the *Il‐10^−/−^* colitis mouse model. Each mouse was gavaged with *O. splanchnicus* at a concentration of 10^8^ CFU/200 µL three times a week for 13 weeks. *O. splanchnicus* was suspended in sterile PBS, and an equivalent volume of sterile PBS was used as the vehicle control. After the intervention with *O. splanchnicus*, weight loss was recorded, the distal colon was collected for HE staining, and the remaining colon was reserved for further analysis. O) Weight loss, P) representative HE staining images at 20× and 40× magnification, and Q) colonization of *O. splanchnicus* in the two groups. Data are presented as mean ± SEM and analyzed by the Mann‐Whitney U test. R) Representative IF staining images for intestinal barrier proteins ZO‐1, CLAUDIN1, and MUC2. S) Statistical analysis of intensity in groups. Data are presented as mean ± SEM. ^****^, *P* < 0.0001, as determined by the nonparametric Kruskal–Wallis test. DSS, dextran sulfate sodium; *O. splanchnicus*, *Odoribacter splanchnicus*; HC, healthy control; UC, ulcerative colitis; ZO‐1, zonula occludens‐1; MUC2, mucin 2; IHC, immunohistochemistry.

We initially observed the correlation between *O. splanchnicus* and patients with UC using 16S rRNA sequencing. The abundance of *O. splanchnicus* (**Figure** **1**A) and the *Odoribacter* genus (Figure S1A, Supporting Information) was reduced in patients with UC, confirming the depletion of *O. splanchnicus* in these individuals. In addition, the *Odoribacter* genera were consistently reduced in both acute and chronic colitis mouse models compared to the control group (Figure S1B, Supporting Information). To further investigate this observation, we assessed the absolute abundance of *O. splanchnicus* in samples of 15 newly diagnosed patients with UC via quantitative PCR (qPCR). The results confirmed the depletion of *O. splanchnicus* in these patients (Figure S1B, Supporting Information). To assess the association with treatment response, we reanalyzed *O. splanchnicus* abundance in fecal samples from patients with UC after FMT treatment, as reported in our previous study.^[^ ^17^ ^]^ The data showed that IgA‐coated *O. splanchnicus* abundance was increased in the treatment responsive group after FMT (Figure S1C, Supporting Information). In summary, *O. splanchnicus* was depleted in colitis, and its abundance was associated with the efficacy of clinical treatments.

### 2.2 *O. splanchnicus* Alleviates Inflammation and Gut Barrier Injury in DSS‐Induced Colitis and Il‐10^−/−^ Mice

The role of *O. splanchnicus* in inflammation and barrier injury repair in colitis was initially evaluated using a DSS‐induced colitis mouse model. We established a 2.5% DSS‐induced acute colitis model and administered *O. splanchnicus* via gavage intervention (Figure 1C). Treatment with *O. splanchnicus* significantly reduced weight loss (Figure 1D). Additionally, the inflammation‐induced shortening of colon length was mitigated (Figure 1H). Histologically, inflammation‐related manifestations of colitis, including mucosal damage, loss of crypts, and immune cell infiltration, were ameliorated (Figure 1E,F). These results demonstrate the protective effect of *O. splanchnicus* in acute colitis.

To detect the colonization of *O. splanchnicus*, we employed qPCR to measure its absolute abundance. The data indicated a significant increase in *O. splanchnicus* in the *O. splanchnicus*‐treated group (Figure 1G). In addition, scanning electron microscopy (SEM) indicated the presence of *O. splanchnicus* on the intestinal surface of both germ‐free mice and antibiotic cocktails (Abx)‐pretreated mice (Supplementary Figure S2A–C), further supporting the colonization of *O. splanchnicus* in the colon.

Gut barrier injury is a critical pathophysiological stage in the development of colitis. To assess changes in the gut barrier, we stained proteins associated with mucosal barriers in the colon of *O. splanchnicus*‐treated mice. The results showed that *O. splanchnicus* upregulated the expression of the tight junction proteins Zo‐1 and Claudin‐1 (Figure 1I–L), as well as the expression of the mucosal protein Muc2 and the number of goblet cells, as observed through Alcian Blue‐Phosphoric Acid Schiff (AB‐PAS) staining (Figure 1K,M). Additionally, consistent results were observed for gut barrier expression at the mRNA level (Figure S2D–F). In summary, *O. splanchnicus* alleviated gut barrier injury, including both the mechanical and mucosal barriers.

Another *Il‐10^−/−^* spontaneous colitis model was used to assess the effect of *O. splanchnicus* on chronic inflammation (Figure 1N). Starting from week 17, *O. splanchnicus* treatment decreased weight loss in mice (Figure 1O). Histologically, hematoxylin and eosin (HE)‐stained images showed a reduction in inflammatory cell infiltration and mucosal damage (Figure 1P). Additionally, we observed a protective effect on the gut barrier following *O. splanchnicus* intervention (Figure 1R,S; Figure S2G–I, Supporting Information). Notably, the abundance of *O. splanchnicus* did not change significantly in the *O. splanchnicus*‐treated group (Figure 1Q), indicating that *O. splanchnicus* may exert an anti‐inflammatory effect without the need for colonization. Taken together, these results highlight the potential of *O. splanchnicus* in reducing inflammation and protecting the gut barrier in colitis.

### 2.3 *O*. *s* *planchnicus* Modulates the Colonic Immune Landscape of Colitis Mice, Especially Neutrophils

**Figure 2:**
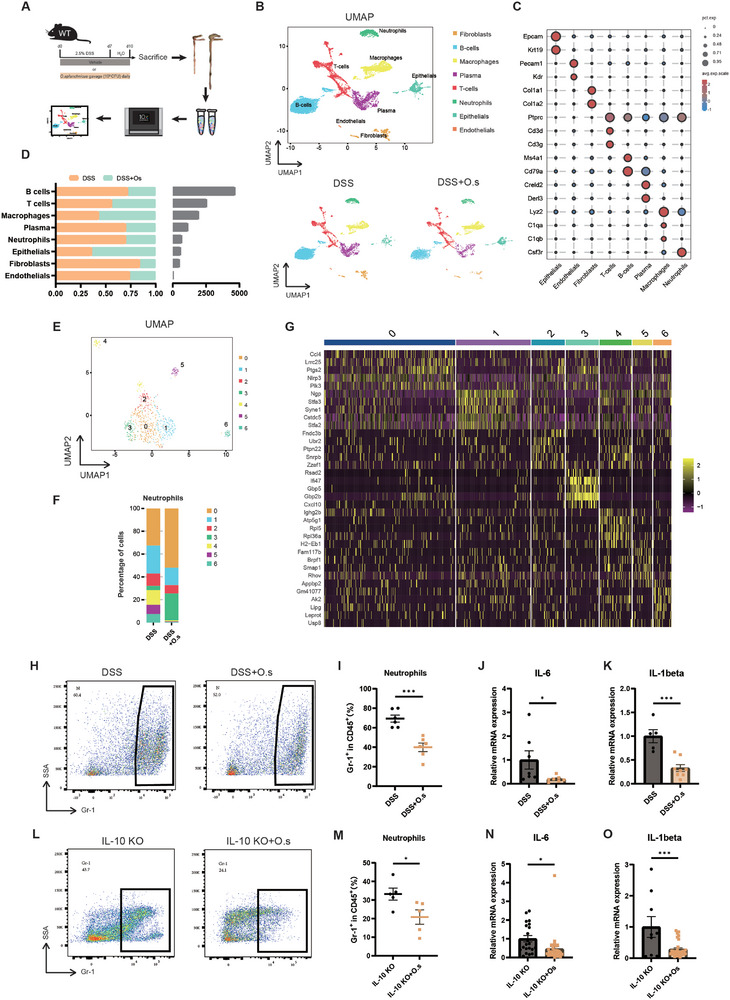
*O. splanchnicus* induces immune landscape remodeling and inhibits neutrophil infiltration in colitis mice. A) Experimental design for single‐cell RNA sequencing in colitis mice treated with *O. splanchnicus*. B) UMAP plot displaying the results for eight cell types. Subpopulations of fibroblasts, B cells, macrophages, plasma cells, T cells, neutrophils, epithelial cells, and endothelial cells in both groups are represented with distinct colors. C) Expression of marker genes in different cell types. The size of the dots indicates the percentage of cells expressing the gene, while the color of the dots reflects the expression level. D) Proportion and absolute cell counts of cell types in the colon of colitis mice treated with *O. splanchnicus*. E) UMAP plots of neutrophils from the colon of colitis mice treated with *O. splanchnicus*. F) Percentage of neutrophil subpopulations in the colon of colitis mice treated with *O. splanchnicus*. G) Heatmap of signature genes for neutrophil clusters. Each cluster is represented by five specifically expressed genes. H) Representative flow cytometry plots and I) statistical analysis of Gr‐1^+^ neutrophils in DSS‐induced colitis mice with or without *O. splanchnicus* treatment. Data are shown as mean ± SEM (n = 6). ^***^, *P* < 0.001 by unpaired Student's t test. J) Relative expression of mRNA for the pro‐inflammatory cytokine Il‐6 and K) Il‐1β decreased in colitis mice following *O. splanchnicus* treatment. Data are shown as mean ± SEM (n = 6). ^*^, *P* < 0.05 by unpaired Student's t test. L) Representative flow cytometry plots and M) statistical analysis of Gr‐1^+^ neutrophils in Il‐10^−/−^ mice with or without *O. splanchnicus* treatment. Data are shown as mean ± SEM (n = 6). ^*^, *P* < 0.05 by unpaired Student's t test. N) Relative expression of mRNA for the pro‐inflammatory cytokine *Il‐6* and O) *Il‐1β* decreased in *O. splanchnicus*‐treated *Il‐10^−/−^* mice. Data are shown as mean ± SEM (n = 6). ^*^, *P* < 0.05; ^***^, *P* < 0.001 as determined by unpaired Student's t test. *O. splanchnicus*, *Odoribacter* s*planchnicus*.

Injury to the gut barrier leads to the entry of large amounts of antigens into the colonic mucosa, leading to an imbalanced immune response in the host. We hypothesized that the protective effects on anti‐inflammatory responses and the gut barrier induced by *O. splanchnicus* are mediated through its interaction with colonic immune cells. To comprehensively explore the underlying immunomodulatory properties of *O. splanchnicus* in colitis, we conducted single‐cell RNA sequencing on colon tissue from *O. splanchnicus*‐treated and vehicle mice (**Figure** **2**A). After quality control, a total of 5495 cells from the *O. splanchnicus*‐treated group and 9629 cells from the control group were included in the subsequent analysis. The unique molecular identifiers (UMI) per cell were 5653.65 for the *O. splanchnicus*‐treated group and 4024.43 for the control group. Using classical cell markers, we classified all analyzed cells into eight groups by uniform manifold approximation and projection (UMAP) (Figure 2B; Figure S3A, Supporting Information), including fibroblasts (*Col1a1*, *Col1a2*), T cells (*Ptprc*, *Cd3d*, *Cd3g*), macrophages (*Lyz2*, *C1qa*, *C1qb*), epithelial cells (*Epcam*, *Krt19*), B cells (*Cd79a*, *Ms4a1*), neutrophils (*Csf3r*), plasma cells (*Creld2*, *Derl3*), and endothelial cells (*Pecam1*, *Kdr*) (Figure 2C). These cell types were detected across all groups. Neutrophils are the first line of defense against antigens and play a role in anti‐inflammation during inflammatory injury.^[^ ^18^ ^]^ Our data suggested that the percentage of neutrophils was decreased in the *O. splanchnicus*‐treated group (Figure 2D). This notable finding warrants further investigation.

To understand the role of *O. splanchnicus* in inflammation, we analyzed the transcriptional and functional changes of neutrophils following *O. splanchnicus* intervention. Using single‐cell RNA sequencing, we categorized the neutrophils into seven clusters (Figure 2E). A decrease in the percentage was observed in clusters 1, 2, 4, 5, and 6, while clusters 0 and 3 were enriched following *O. splanchnicus* intervention (Figure 2F). Gene and pathway enrichment analysis indicated that the upregulated genes in Cluster 0 (e.g., *Ccl4*, *Lrrc25*, *Ptgs2*, *Nlrp3*, *Plk3*) (Figure 2G) were associated with the NOD‐like receptor signaling pathway, TNF signaling pathway, and chemokine signaling pathway (Figure S3B). In Cluster 1, the upregulated genes (e.g., *Ngp*, *Stfa3*, *Syne1*, *Cstdc5*, *Stfa2*) (Figure 2G) were associated with apoptosis (Figure S3C). Cluster 2 showed upregulated genes (e.g., *Fndc3b*, *Ubr2*, *Ptpn22*, *Snrpb*, *Zzef1*) (Figure 2G) associated with the C‐type lectin receptor signaling pathway and oxidative phosphorylation (Figure S3D, Supporting Information). Finally, in Cluster 3, the upregulated genes (e.g., *Rsad2*, *Ifi47*, *Gbp5*, *Gbp2b*, *Cxcl10*) (Figure 2G) were associated with autophagy, TNF signaling, and mitophagy (Figure S3E).

**Figure 3:**
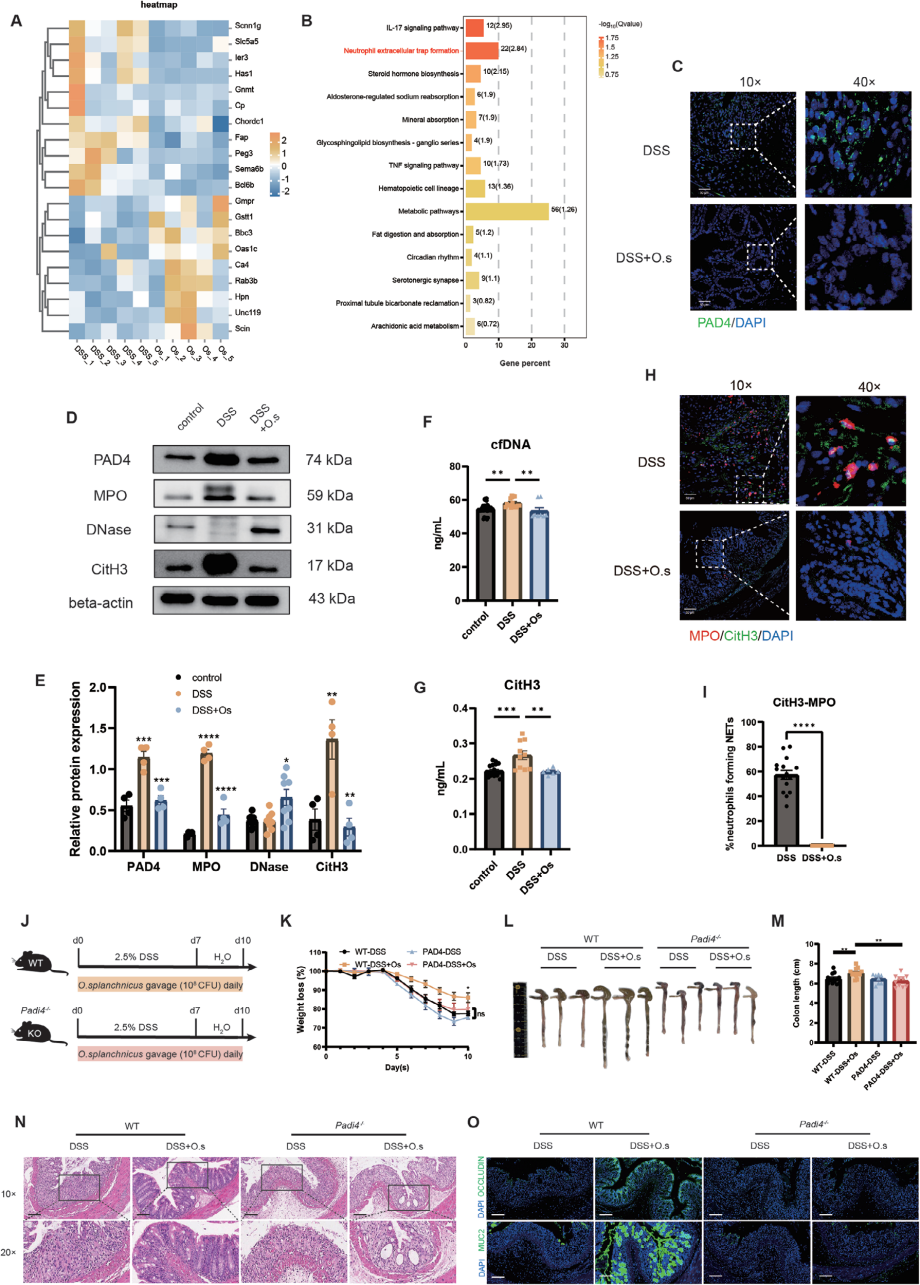
*O. splanchnicus* exerts anti‐inflammatory and gut barrier‐protective effects by regulating the formation and degradation of NETs. A) Heatmap of differential gene expression in colitis mice with or without *O. splanchnicus* intervention. B) KEGG pathway enrichment analysis of differentially expressed genes in *O. splanchnicus*‐treated colitis mice. C) Representative IF images showing Pad4 (green) and DAPI (blue) staining at 10× and 40× magnification. Scale bar, 50 µm. D) Western blot analysis of Pad4, MPO, DNase, and CitH3 in the colon from control and *O. splanchnicus*‐treated groups, with β‐actin as the loading control. E) Statistical analysis of western blot data for Pad4, MPO, DNase, and CitH3 expression, compared to β‐actin in the three groups. Data are presented as mean ± SEM (n = 8 for DNase, n = 4 for the other indexes). ^*^, *P* < 0.05; ^**^, *P* < 0.01; ^***^, *P* < 0.001; ^****^, *P* < 0.0001, as determined by nonparametric Kruskal–Wallis test. F) ELISA results for the concentration of cell‐free DNA. G) ELISA results for citrullinated histone H3 levels. H) Representative IF images of CitH3 (green) and Mpo (red), as well as CitH3 (green) and NE (red). Scale bar, 50 µm. I) Statistical analysis of the percentage of NETs‐forming neutrophils in IF images co‐stained with CitH3 and MPO. Data are presented as mean ± SEM. ^****^, *P* < 0.0001, as determined by nonparametric Kruskal–Wallis test. J) Experimental design for the DSS‐induced colitis model in wild‐type and *Pad4^−/−^* mice. Mice were treated with 2.5% DSS in drinking water for 7 days, followed by 3 days of recovery with normal water. Each mouse was orally gavaged daily with 10^8^ CFU/200 µL *O. splanchnicus* for 10 days. At the end of the experiment, mice were euthanized. Representative images of colonic appearance are shown. The distal colon was fixed in 4% paraformaldehyde, and sections were stained with HE. K) Weight loss data. L) Representative images of colon morphology. M) Statistical analysis of colon length. Data are presented as mean ± SEM (n = 10). ^*^, *P* < 0.05; ^**^, *P* < 0.01, as determined by nonparametric Kruskal–Wallis test. N) Representative HE staining images in wild‐type and *Pad4^−/−^* mice following *O. splanchnicus* intervention. O) Representative IF images of Occludin and Muc2 staining in wild‐type and *Pad4^−/−^* mice after *O. splanchnicus* intervention. DSS, dextran sulfate sodium; cfDNA, double‐strain DNA; *O. splanchnicus*, *Odoribacter* s*planchnicus*; Muc2, mucin 2.

To verify the reduction of neutrophils following *O. splanchnicus* intervention, we assessed the frequency of Gr‐1^+^ neutrophils in DSS‐induced colitis and *Il‐10^−/−^* mouse models via flow cytometry. The percentage of Gr‐1^+^ cells within the CD45^+^ cell population markedly decreased in the *O. splanchnicus*‐treated group (**Figure** **3**H,I). Additionally, *O. splanchnicus* downregulated the expression of the pro‐inflammatory cytokines *Il‐6* and *Il‐1β* (Figure 3J,K). Consistent results were observed in the *Il‐10^−/−^* mice (Figure 3L–O). To evaluate whether neutrophils were required for the anti‐inflammatory effect of *O. splanchnicus*, we performed in vivo neutrophil depletion using an anti‐Ly6G antibody. Mice were injected intraperitoneally at day −1, 1, 3, and 5 during the course of DSS‐induced colitis (Figure S4A). Compared with the isotype control group, the anti‐inflammatory effect of *O. splanchnicus* was abolished in neutrophil‐depleted mice (Figure S4B–D, Supporting Information), suggesting that neutrophils are essential for mediating this protective effect. In summary, *O. splanchnicus* modulated the immune landscape of the colon under inflammatory conditions, and neutrophils might be involved in its protective effect in both acute DSS‐induced colitis and *Il‐10^−/−^* spontaneous colitis mouse models.

### 2.4 *O*. *s* *planchnicus* Exerts Anti‐Inflammatory and Gut Barrier‐Protective Effects by Regulating the Formation and Degradation of NETs

To identify genes regulated by *O. splanchnicus* in colitis mice, we performed bulk transcriptome sequencing and differential gene analysis on colon samples from *O. splanchnicus*‐treated and control groups. The volcano plot showed differential genes following *O. splanchnicus* intervention (Figure S5A, Supporting Information), including 255 upregulated genes and 269 downregulated genes. The heatmap showed the top differentially expressed genes between the two groups (Figure 3A). Kyoto Encyclopedia of Genes and Genomes (KEGG) analysis showed the enrichment of multiple inflammatory and immune pathways, particularly the NETs formation pathway (Figure 3B), which is associated with neutrophil function. The process of NETs formation, also known as NETosis, is characterized by the release of web‐like structures consisting of decondensed DNA and antimicrobial proteins, including MPO and NE.^[^ ^8a^ ^]^

For verification, we first examined the single‐cell RNA sequencing data, which suggested that CD177, a gene associated with NETs formation, is expressed in all neutrophil clusters (Figure S5B, Supporting Information). *O. splanchnicus* decreased the expression of *Cd177*, as demonstrated by UMAP analysis (Figure S5C, Supporting Information). The proteins associated with NETs formation were then validated. PAD4 is a key enzyme that promotes histone citrullination (CitH3), which in turn facilitates histone decondensation and CitH3 release.^[^ ^19^ ^]^ The expression of Pad4 was significantly decreased in the *O. splanchnicus*‐treated group, as observed by immunofluorescence (IF) staining (Figure 3C,H; Figure S5D, Supporting Information) and western blot (Figure 3D,E). Furthermore, *O. splanchnicus* downregulated the levels of MPO and CitH3 proteins released from neutrophils, as shown by western blot analysis (Figure 3D,E). The levels of CitH3 and cell‐free DNA (cfDNA) in the serum were also measured by enzyme‐linked immunosorbent assay (ELISA), which showed that *O. splanchnicus* significantly reduced the levels of CitH3 and cfDNA induced by inflammation (Figure 3F,G). The IF images showed the colocalization of citrullinated histones with released antimicrobial proteins from neutrophils. Compared to the DSS group, *O. splanchnicus* intervention reduced the colocalization of CitH3 with MPO (Figure 3H,I). In addition, DNase facilitates the degradation of NETs released by neutrophils.^[^ ^12^ ^]^ Our data suggested that DNase expression was elevated in the *O. splanchnicus*‐treated group, suggesting that NET degradation might be enhanced by *O. splanchnicus* (Figure 3D,E). Overall, *O. splanchnicus* modulated neutrophil function by decreasing the formation of NETs and pro‐inflammatory contents while promoting NETs degradation.

To assess whether *O. splanchnicus* plays a protective role in inhibiting inflammation in NETs‐deficient *Pad4^−/−^* mice, we established an acute colitis model using 2.5% DSS in *Pad4^−/−^* mice to examine the effect of *O. splanchnicus* on inflammation (Figure 3J). Treatment with *O. splanchnicus* in *Pad4^−/−^* mice did not alleviate weight loss, colon shortening, colonic histological damage, or barrier disruption (Figure 3K–O; Figure S5E, Supporting Information). Overall, these results suggested that *O. splanchnicus* exerts anti‐inflammatory and gut barrier‐protective effects by modulating PAD4 to regulate the formation and degradation of NETs.

### 2.5 *O*. *s* *planchnicus* Reshapes the Bile Acid Pool to Promote LCA‐Driven Neutrophil Modulation

Commensal bacteria have been reported to regulate mucosal immunity through metabolites or interactions with the gut microbiota. To assess whether the membrane proteins of *O. splanchnicus* have effects similar to those of live *O. splanchnicus*, we evaluated the anti‐inflammatory properties of heat‐killed *O. splanchnicus* (Figure S6A). Treatment with heat‐killed *O. splanchnicus* failed to alleviate inflammation and barrier damage (Figure S6B–G, Supporting Information). These results indicated that membrane proteins alone were insufficient to inhibit colitis. To explore the characteristics of anti‐inflammatory components of live *O. splanchnicus*, we gavaged DSS‐induced colitis mice with phosphate‐buffered saline (PBS), conditional medium of *O. splanchnicus* (CM), and conditional medium of *O. splanchnicus* treated with proteinase K (CMK), which removes enzymes and other protein components (Figure S7A, Supporting Information). Results showed that treatment with either CM or CMK effectively alleviated inflammation and preserved epithelial barrier integrity. (Figure S7B–H, Supporting Information). To further determine whether small molecular metabolites or non‐protein components of live *O. splanchnicus* are required for protection, we separated the conditional medium of *O. splanchnicus* into >3 kDa and <3 kDa fractions based on molecular weight (Figure S8A, Supporting Information). We found that only the <3 kDa fraction of *O. splanchnicus* supernatant exhibited anti‐inflammatory properties and was able to repair gut barrier damage in colitis mice (Figure S8B–H, Supporting Information), which suggested small‐molecule metabolites present in the conditioned medium might mediated the protection of *O. splanchnicus*. To investigate metabolite alterations in the conditioned medium of *O. splanchnicus*, Untargeted metabolomic profiling was performed, and identified a significant increase in indole‐3‐acetic acid, indole‐3‐acrylic acid, and indole‐3‐pyruvic acid (Figure S9A–C, Supporting Information). Taken together, these findings suggested that the interaction with the host is primarily mediated by live *O. splanchnicus* and its metabolites, rather than by membrane proteins or enzymatic components in the supernatant.

**Figure 4:**
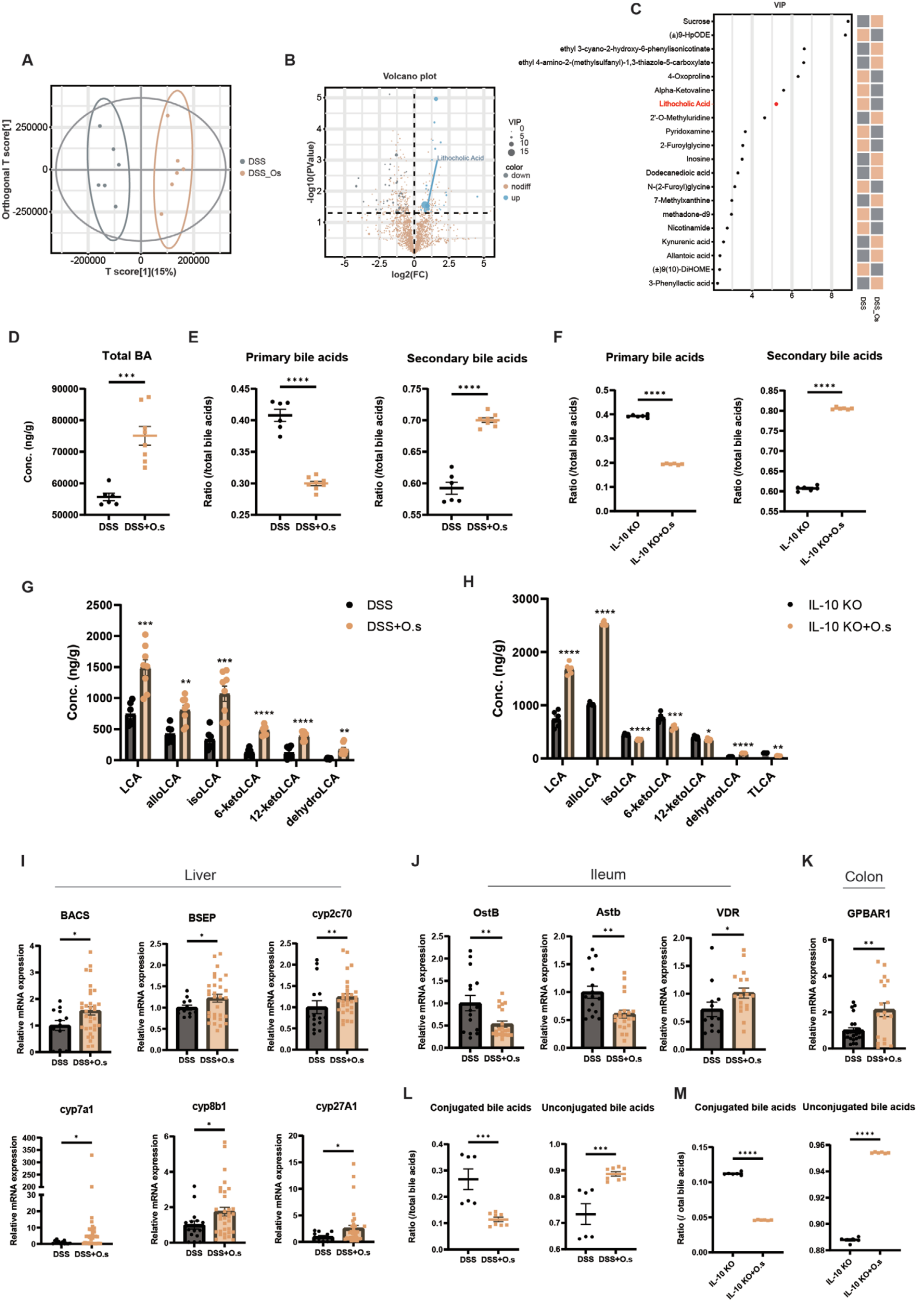
*O. splanchnicus* modulates bile acid metabolism in DSS‐induced colitis and *Il‐10^−/−^* mouse models. A) OPLS‐DA revealed distinct metabolic profiles between the two groups through LC‐MS/MS. B) The volcano plot shows differential metabolites between the two groups. The size of the dots represents the VIP scores, with blue dots indicating increased metabolites and gray dots indicating decreased metabolites. C) Differential metabolites are shown based on VIP scores, with LCA being enriched following *O. splanchnicus* intervention. D) LC‐MS analysis of the total concentration of bile acid in feces of DSS‐induced colitis mice following *O. splanchnicus* intervention. E) The ratio of primary and secondary bile acid relative to total bile acid in DSS‐colitis mice with *O. splanchnicus* intervention. F) The ratio of primary and secondary bile acid relative to total bile acid in *Il‐10^−/−^* mice with *O. splanchnicus* intervention. G) Concentrations of LCA and its derivatives in feces following *O. splanchnicus* treatment in DSS‐colitis mice. H) Concentrations of LCA and its derivatives in feces of Il‐10^−/‐^ mice after *O. splanchnicus* treatment. Data are shown as mean ± SEM. ^*^, *P* < 0.05; ^**^, *P* < 0.01; ^***^, *P* < 0.001; ^****^, *P* < 0.0001, as determined by Mann–Whitney U test. I) The mRNA expression of bile acid synthesis‐related genes (*Bacs*, *Bsep*, *Cyp2c70*, *Cyp7a1*, *Cyp8b1*, and *Cyp27a1*) in the liver of mice treated with *O. splanchnicus*. J) The mRNA expression of bile acid synthesis‐related genes (*OstB*, *Vdr*, *Astb*) in the ileum of mice treated with *O. splanchnicus*. K) The mRNA expression of bile acid synthesis‐related genes (*Gpbar1*) in the colon of mice treated with *O. splanchnicus*. Data are shown as mean ± SEM. ^*^, *P* < 0.05; ^**^, *P* < 0.01, by Mann–Whitney U test. L) The ratio of conjugated and unconjugated bile acid to total bile acid in feces of DSS‐induced colitis and M) *Il‐10^−/‐^* mice after *O. splanchnicus* treatment. Data are shown as mean ± SEM. ^*^, *P* < 0.05; ^**^, *P* < 0.01; ^***^, *P* < 0.001; ^****^, *P* < 0.0001, by Mann–Whitney U test.

To identify potential metabolites affected by *O. splanchnicus*, we conducted untargeted metabolomic sequencing of the feces from colitis mice using liquid chromatography‐tandem mass spectrometry (LC‐MS/MS). Orthogonal projections to latent structures discriminant analysis (OPLS‐DA) revealed distinct metabolic profiles between the two groups (**Figure** **4**A). Differential metabolites were identified based on variable importance in projection (VIP) scores (Figure 4B,C). LCA, a secondary bile acid, was significantly enriched following *O. splanchnicus* intervention (Figure 4C). Targeted metabolomic analysis of bile acids indicated a significant increase in total bile acid concentration in the feces following *O. splanchnicus* intervention (Figure 4D). Compared to the DSS group, the *O. splanchnicus*‐treated group exhibited an increased ratio of secondary to primary bile acids (Figure 4E). Notably, the levels of LCA and its derivatives were elevated in the feces (Figure 4G). The consistent results of increased secondary bile acids and LCA levels can be observed in another *Il‐10^−/−^* mice model after *O. splanchnicus* intervention (Figure 4F,H). To verify the effect of *O. splanchnicus* on bile acid metabolism, we analyzed the expression of genes involved in bile acid metabolism, synthesis, and excretion in the liver, ileum, and colon using qPCR (Figure 4I–K; Figure S10A–C, Supporting Information). In the liver, the expression of *Bacs*, *Bsep*, *Cyp2c70*, *Cyp7a1*, *Cyp8b1*, and *Cyp27a1* was upregulated after *O. splanchnicus* intervention (Figure 4I). Moreover, *O. splanchnicus* downregulated the expression of bile acid transporters *Ostb* and *Astb*, while upregulating the expression of *Vdr* in the ileum (Figure 4J). An increased expression of the bile acid receptor *Gpbar1* was also observed in the colon of the *O. splanchnicus*‐treated group (Figure 4K). The transformation and production of secondary bile acids involve enzymes from the gut microbiota, which convert primary bile acids excreted into the intestine into secondary bile acids through deconjugation reactions and subsequent dihydroxylation.^[^ ^20^ ^]^ Consistently, conjugated bile acids were downregulated, while unconjugated bile acids were upregulated in the feces of the *O. splanchnicus*‐treated group (Figure 4L,M). To assess whether *O. splanchnicus* can regulate the bile acid‐metabolizing microbiota in colitis mice, we analyzed the composition of the gut microbiota using 16S rRNA sequencing. *O. splanchnicus* increased the diversity of the gut microbiota (Figure S11A,B, Supporting Information), and specific potential probiotics, including *Bacteroides*, *Oscillibacter*, *Alistipes*, and *Muribaculum*, were enriched in the *O. splanchnicus*‐treated group (Figure S11C, Supporting Information). In addition, we observed changes in the expression of genes regulating bile acid metabolism in *Il‐10^−/−^* mice (Figure S10D–F; Figure S11D–F, Supporting Information). Collectively, these results suggested that *O. splanchnicus* significantly reshaped the secondary bile acid profile, with the most pronounced effects on LCA accumulation, in two experimental colitis models.

**Figure 5:**
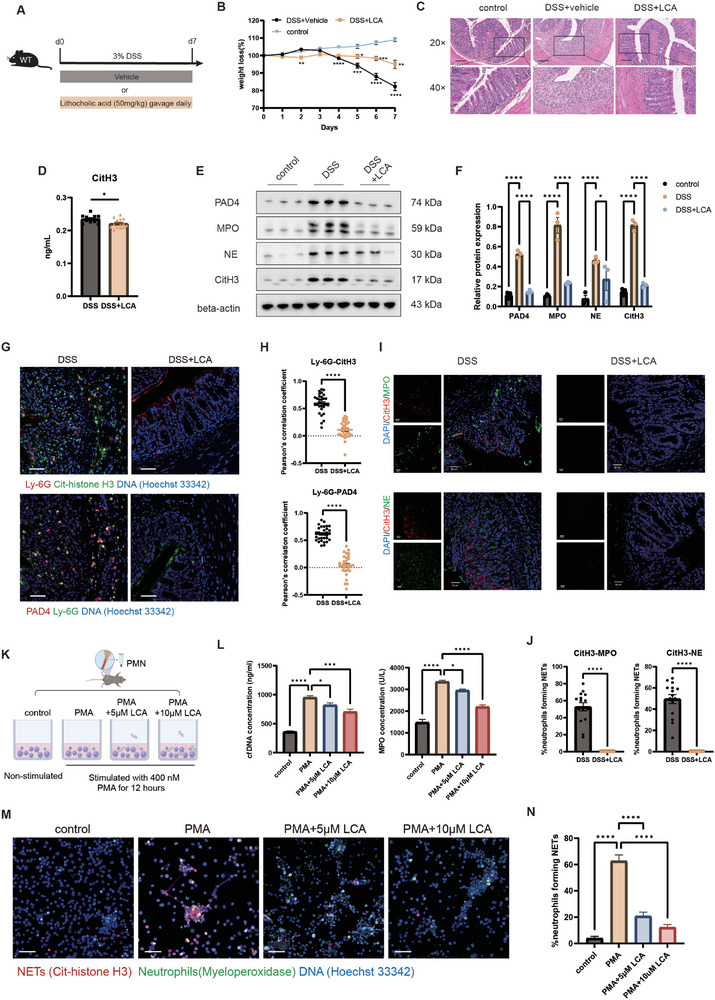
*O. splanchnicus*‐mediated LCA inhibits NET formation. A) Experimental design of 3% DSS‐induced colitis mice treated with 50 mg/kg LCA for 7 days. B) Weight loss in the three groups. C) Representative images of HE staining at 20× and 40× magnification. D) ELISA results for detecting CitH3 concentrations after LCA treatment. E) Western blot analysis and F) the statistical analysis of PAD4, MPO, NE, and CitH3 in the colon from control and LCA‐treated groups, using β‐actin as the loading control. G) Representative images of IF staining for CitH3 (green) and Ly6G (red), Ly6G (green) and PAD4 (red). Hoechst 33342 was used to stain nuclei (blue). H) The colocalization between Ly‐6G with CitH3 or PAD4 was quantified using Pearson's correlation coefficient (PCC). PCC values were calculated from five fields per sample (n = 3 mice/group). Data represent the mean percentage of NET forming neutrophils from three mice per group, with five fields analyzed per sample. Data are shown as mean ± SEM (n = 15). ^****^, *P* < 0.0001, as determined by the unpaired Student's t test. I) Representative images of IF staining for CitH3 (red) and MPO (green), CitH3 (red) and NE (green). DAPI was used to stain nuclei (blue). Scale bar, 50 µm. J) The percentage of NETs forming neutrophils in the two groups. Data represent the mean percentage of NET forming neutrophils from three mice per group, with five fields analyzed per sample. Data are shown as mean ± SEM (n = 15). ^****^, *P* < 0.0001, as determined by the unpaired Student's t test. K) Experimental design of isolated murine neutrophils from bone marrow stimulated with 400 nM PMA and treated with LCA at 5 µM and 10 µM concentration. L) ELISA results for detecting cfDNA AND MPO concentration. M) Representative IF images of colocalization between CitH3 (red) and MPO (green). Hoechst 33342 was used to stain nuclei (blue). N) Statistical analysis of the percentage of NETs forming neutrophils.

LCA is a major secondary bile acid. We hypothesized that the upregulation of LCA is the mechanism through which *O. splanchnicus* exerts its protective role in alleviating inflammation and barrier damage. To determine whether LCA supplementation alone is sufficient to alleviate inflammation and barrier injury in colitis, we gavaged DSS‐induced colitis mice with LCA (**Figure** **5**A). The results revealed that LCA similarly alleviated weight loss, colonic shortening, and histological injury (Figure 5B,C; Figure S12A–C, Supporting Information), including crypt damage, pro‐inflammatory immune cell infiltration, and barrier damage (Figure S12D, Supporting Information). To assess whether LCA supplementation similarly modulates the colonic immune cell microenvironment, we examined the number and function of colonic neutrophils in colitis mice gavaged with LCA. Consistently, the results showed that LCA reduced Gr‐1^+^ neutrophil infiltration in the colonic tissue (Figure S12E,F, Supporting Information) and the expression of pro‐inflammatory cytokines *Il‐1β* and *Il‐6* (Figure S12G, Supporting Information). The expression of NETs formation‐related proteins PAD4, MPO, NE, and CitH3 detected by western blot, were downregulated after LCA intervention (Figure 5E,F). In addition, LCA reduced the level of CitH3 in serum (Figure 5D). Moreover, decreased NETs forming neutrophils was also observed in the LCA‐treated group, as evidenced by costaining of citrullinated histones 3 with MPO and NE (Figure 5I,J). The consistent trends showed by less colocalization of Ly6G with CitH3 and PAD4 (Figure 5G,H).

To determine whether LCA has a direct inhibitory effect on NETs formation, we conducted a coculture experiment in vitro by using primary murine neutrophils (Figure 5K). The results showed that LCA significantly inhibited NETs formation, as evidenced by decreased CitH3 and MPO colocalization (Figure 5M,N) and less extracellular web‐like DNA structure (Figure S12H, Supporting Information). The quantification results of ELISA also demonstrated decreased levels of cfDNA and MPO (Figure 5L). Interestingly, the above inhibitory role on NETs formation was not observed after the conditioned medium of *O. splanchnicus* intervention (Figure S13A–C, Supporting Information). Taken together, these data demonstrated that *O. splanchnicus* triggered a gut microbiota‐related, secondary bile acid metabolism‐driven modulation of neutrophil function, thereby exerting a protective effect in inhibiting inflammation and promoting gut barrier repair.

### 2.6 Promotion of LCA Transformation by *O*. *s* *planchnicus* is Dependent on Gut Microbiota

**Figure 6:**
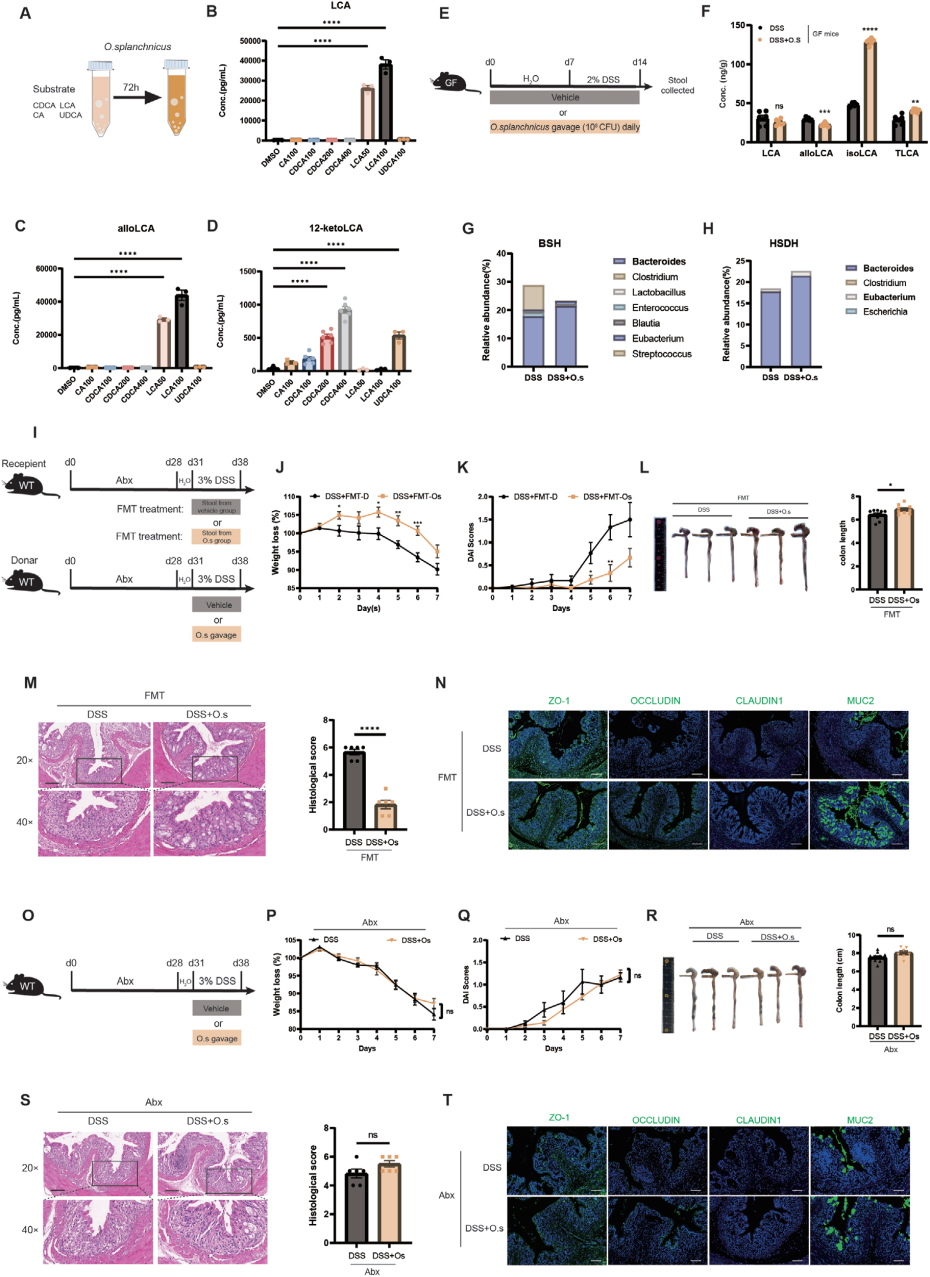
*O. splanchnicus* promotes LCA transformation via gut microbiota. A) Experimental design for in vitro culture of *O. splanchnicus*. *O. splanchnicus* (OD = 0.1) was supplemented with CA, CDCA, LCA, and UDCA. The conditioned medium was analyzed for the concentration of bile acid. B) Concentrations of LCA, C) alloLCA, and D) 12‐ketoLCA. Data are shown as mean ± SEM. ^****^, *P* < 0.0001, as determined by the nonparametric Kruskal–Wallis’ test. E) Experimental design for germ‐free mice treated with *O. splanchnicus*. Each mouse was gavaged daily with *O. splanchnicus* at a concentration of 10^8^ CFU/200 µL for 2 weeks. In the final week of the experiment, 2% DSS was added to the drinking water. *O. splanchnicus* was suspended in sterile PBS, and an equivalent volume of sterile PBS was used as a vehicle control. F) Concentrations of LCA and its derivatives in the feces of germ‐free mice. Data are shown as mean ± SEM. ^**^, *P* < 0.01; ^***^, *P* < 0.001; ^****^, *P* < 0.0001, as determined by unpaired Student's t test. G) Relative abundance of BSH‐containing genera in DSS‐induced colitis mice following *O. splanchnicus* intervention. H) Relative abundance of HSDH‐containing genera in DSS‐induced colitis mice following *O. splanchnicus* intervention. I) Experimental design for 3% DSS‐induced colitis mice receiving FMT. Mice were pretreated with Abx for 4 weeks to deplete most bacteria in the gut. Donor mice received *O. splanchnicus*‐containing stool via oral gavage, while also being treated with 3% DSS in drinking water. Weight loss, DAI, and colon length were monitored. Distal colon tissue was collected for HE staining, and the remaining colon was reserved for further validation. J) Weight loss, K) DAI scores, L) representative images of the colon, and statistical analysis of colon length. Data are shown as mean ± SEM. ^*^, *P* < 0.05; ^**^, *P* < 0.01; ^***^, *P* < 0.001, as determined by unpaired Student's t test. M) Representative images of HE staining at 20× and 40× magnification, with comparison of statistical histological scores across groups. Data are shown as mean ± SEM. ^****^, *P* < 0.0001, as determined by unpaired Student's t test. N) Representative images of IF staining for Zo‐1 (green), Occludin (green), Claudin1 (green), and Muc2 (green) in colitis mice. O) Experimental design for 3% DSS‐induced colitis mice receiving Abx pretreatment. Ampicillin (1 g L^−1^), neomycin (1 g L^−1^), metronidazole (1 g/L), and vancomycin (0.5 g/L) were added to the drinking water of mice ad libitum for 4 weeks to eliminate most gut bacteria. Subsequently, *O. splanchnicus* was administered via oral gavage, and 3% DSS was added to the drinking water for 7 days. P) Weight loss, (Q) DAI scores, (R) representative images of the colon, and statistical analysis of colon length. Data are shown as mean ± SEM. “ns” indicates no significant difference, as determined by unpaired Student's t test. S) Representative images of HE staining at 20× and 40× magnification, with comparison of statistical histological scores across groups. T) Representative images of IF staining for Zo‐1 (green), Occludin (green), Claudin1 (green), and Muc2 (green) in colitis mice with Abx pretreatment.

We next investigated how bile acid metabolism mediated by *O. splanchnicus* inhibits inflammation and promotes gut barrier repair. Some taxa within the *Odoribacter* genus can transform secondary bile acids and their derivatives in the host.^[^ ^21^ ^]^ However, whether *O. splanchnicus* exerts a similar effect remains unclear. To determine if *O. splanchnicus* directly induces the transformation of LCA, we supplemented its conditioned medium with primary bile acid substrates for 72 h (**Figure** **6**A). The conditioned medium was then collected for mass spectrometry analysis to measure secondary bile acid levels. Despite substrate supplementation, there was no detectable increase in LCA levels. (Figure 6B). Notably, a concentration‐dependent increase in the levels of the LCA derivatives alloLCA (Figure 6C) and 12‐ketoLCA (Figure 6D) was observed. To further evaluate the transformation of LCA in vivo, we treated germ‐free mice with *O. splanchnicus* orally for 2 weeks (Figure 6E). Mice treated with *O. splanchnicus* did not show increased levels of LCA (Figure 6F). However, the derivative isoLCA was significantly elevated (Figure 6F). Overall, these data indicate that *O. splanchnicus* does not directly transform LCA in vitro or in vivo.

As noted above, *O. splanchnicus* markedly influenced gut microbiota composition, which imply that the presence of diverse gut microbiota may be necessary for *O. splanchnicus* to facilitate the transformation of LCA and its derivatives. Bile salt hydrolase (BSH) and hydroxysteroid dehydrogenase (HSDH) are required by certain bacteria for deconjugation and dihydroxylation.^[^ ^20^ ^]^ Firstly, we analyzed the abundance of genera containing^[^ ^22^ ^]^ BSH and HSDH.^[^ ^23^ ^]^ Notably, the relative abundance of BSH‐containing *Bacteroides* and HSDH‐containing bacteria, including *Bacteroides* and *Eubacterium*, were increased in the *O. splanchnicus*‐treated group (Figure 6G,H). Taken together, the above data suggested the association between gut microbiota composition and bile acid following *O. splanchnicus* intervention.

To explore whether interaction with the altered gut microbiota mediates the protective effects of *O. splanchnicus*, we conducted an FMT experiment to assess inflammation and bile acid levels (Figure 6I). The results showed that the anti‐inflammatory effects of *O. splanchnicus* could be transferred to Abx‐pretreated mice through FMT (Figure 6J–L). Compared to the control group, colonic immune cell infiltration (Figure 6M) and intestinal barrier damage (Figure 6N; Figure S14B, Supporting Information) were alleviated in colitis mice receiving feces from the *O. splanchnicus*‐treated group. Moreover, the levels of LCA and its derivatives were similarly increased in colitis mice receiving feces from the *O. splanchnicus*‐treated group (Figure S15A, Supporting Information). In summary, the altered microbiota mediated the transformation of intestinal LCA by *O. splanchnicus* in mice, suggesting that gut microbiota is essential for secondary bile acid production and the protective effects of *O. splanchnicus*.

Following Abx pretreatment to simulate a microbiota‐deficient condition (Figure 6O), both DSS‐ and *O. splanchnicus*‐treated mice exhibited similar trends in weight loss (Figure 6P), increased DAI scores (Figure 6Q), and colon shortening (Figure 6R). In addition, no differences were observed in histological scores (Figure 6S) or gut barrier repair (Figure 6T; Figure S14A, Supporting Information) in *O. splanchnicus*‐treated mice pretreated with Abx. Notably, the expression of the colonic bile acid transporter, Vdr, was increased in FMT mice (Figure S15C, Supporting Information) but not in germ‐free mice (Figure S15D, Supporting Information) or Abx‐treated mice (Figure S15B, Supporting Information) after *O. splanchnicus* intervention. Collectively, these results suggested that the interaction between *O. splanchnicus* and gut microbiota is necessary for the protective effect of *O. splanchnicus* through the transformation of LCA.

### 2.7 Increased LCA Production by *O*. *s* *planchnicus* Inhibits NET Formation Mediated by Nlrp3‐Gsdmd Signaling

**Figure 7:**
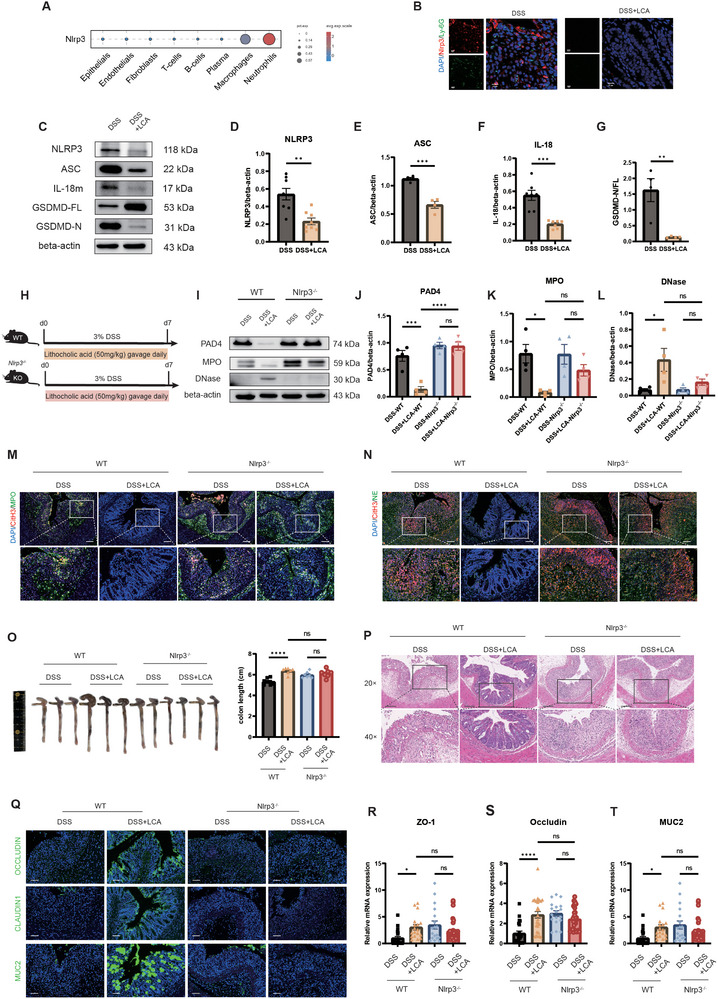
Increased LCA production by *O. splanchnicus* inhibits NET formation mediated by NLRP3‐GSDMD signaling. A) Expression of *Nlrp3* in various cell types following *O. splanchnicus* intervention, as determined by single‐cell RNA sequencing data. B) IF staining showing colocalization of Nlrp3 (red) and Ly‐6G (green), with DAPI used to stain nuclei (blue). Scale bar, 50 µm. C) Western blot analysis of NLRP3, ASC, IL‐18, GSDMD‐FL, and GSDMD‐N in the colon from control and LCA‐treated groups, using β‐actin as a loading control. D–G) Statistical analysis of western blot data performed using ImageJ software. Data are presented as mean ± SEM. ^**^, *P* < 0.01; ^***^, *P* < 0.001, as determined by unpaired Student's t‐test. H) Experimental design for the DSS‐induced colitis model in wild‐type and *Nlrp3^−/−^* mice. Mice were administered 3% DSS in drinking water for 7 days, followed by daily oral gavage of 50 mg kg^−1^ LCA for 7 days. Mice were euthanized at the study's conclusion, and representative images of their colons are shown. The distal of colon were fixed in 4% paraformaldehyde, and sections were stained with HE. I) Western blot analysis of Pad4, MPO, and DNase in the colon from wild‐type and *Nlrp3^−/−^* mice with or without LCA intervention, using β‐actin as a loading control. J–L) Statistical analysis of western blot data performed using ImageJ software. Data are shown as mean ± SEM. ^*^, *P* < 0.05; ^***^, *P* < 0.001, as determined by nonparametric Kruskal–Wallis test. M) Representative IF images showing CitH3 (red) and MPO (green). N) Representative images of CitH3 (red) and Ne (green) in wild‐type and *Nlrp3^−/−^* mice with or without LCA intervention. DAPI was used to stain nuclei (blue). O) Representative images of colon sections and statistical analysis of colon length in the different groups. Data are presented as mean ± SEM. ^****^, *P* < 0.0001, as determined by nonparametric Kruskal–Wallis test. P) Representative HE staining images from wild‐type and *Nlrp3^−/−^* mice following LCA intervention. Q) Representative images of IF staining for Occludin (green), Claudin1 (green), and Muc2 (green) in wild‐type and *Nlrp3^−/−^* mice after LCA intervention. R–T) The mRNA expression levels of barrier‐related genes Zo‐1, Occludin, and Muc2. Data are presented as mean ± SEM. ^*^, *P* < 0.05; ^****^, *P* < 0.0001, as determined by nonparametric Kruskal–Wallis test.

The NLRP3 pathway has been associated with inflammation and immune responses to certain gut microbiota and their metabolites.^[^ ^24^ ^]^ To investigate whether the modulation of NET balance in the colon requires the engagement of NLRP3, we analyzed colon tissue from mice treated with LCA via western blot (**Figure** **7**C). The expression of NLRP3, ASC, and IL‐18 was reduced following LCA interventions (Figure 7C–F). The proportion of cleaved GSDMD, which is associated with pyroptosis, was also decreased (Figure 7C,G). These data indicated that LCA intervention induces a response in the NLRP3 pathway and alters the pyroptosis phenotype. This prompted us to examine previously obtained single‐cell RNA sequencing data for *Nlrp3* expression in immune cells. We confirmed that *Nlrp3* expression was highest in neutrophils and that *Nlrp3* expression was reduced in colonic neutrophils in the *O. splanchnicus*‐treated group (Figure 7A). Moreover, colocalization of NLRP3 and the neutrophil marker Ly‐6G, detected through IF staining (Figure 7B; Figure S16A, Supporting Information), further corroborated this finding.

To investigate whether NLRP3 plays a role in inhibiting NET formation by LCA, we analyzed the regulatory effect of LCA in the absence of the NLRP3 signaling pathway (Figure 7H). Notably, *Nlrp3^−/−^* mice exhibited an absence of the protective effects of LCA on immune environment modulation and inflammation inhibition. The differences in the expression of NET‐related proteins, including PAD4, MPO, and DNase, were no longer observed in *Nlrp3^−/−^* mice after LCA intervention (Figure 7I–L). Moreover, LCA failed to reduce the colocalization of CitH3 with the antimicrobial proteins MPO (Figure 7M; Figure S16B, Supporting Information) and NE (Figure 7N; Figure S16B, Supporting Information) in *Nlrp3^−/−^* mice. These results indicated that the NLRP3 signaling pathway is essential for the inhibition of NET formation by LCA. In addition, no protective effects of LCA or *O. splanchnicus* (Figure S16D,E, Supporting Information) were observed regarding inflammation inhibition (Figure 7O,P) and gut barrier repair (Figure 7Q–T; Figure S16C, Supporting Information) in *Nlrp3^−/−^* mice. Taken together, these results indicated that *O. splanchnicus* promotes the transformation of LCA to modulate NET formation and degradation via the NLRP3‐GSDMD pathway, contributing to a protective effect in colitis mice.

### 2.8 LCA, NETs, and Nlrp3 Expression is Associated with *O*. *s* *planchnicus* Abundance in Patients with UC

**Figure 8:**
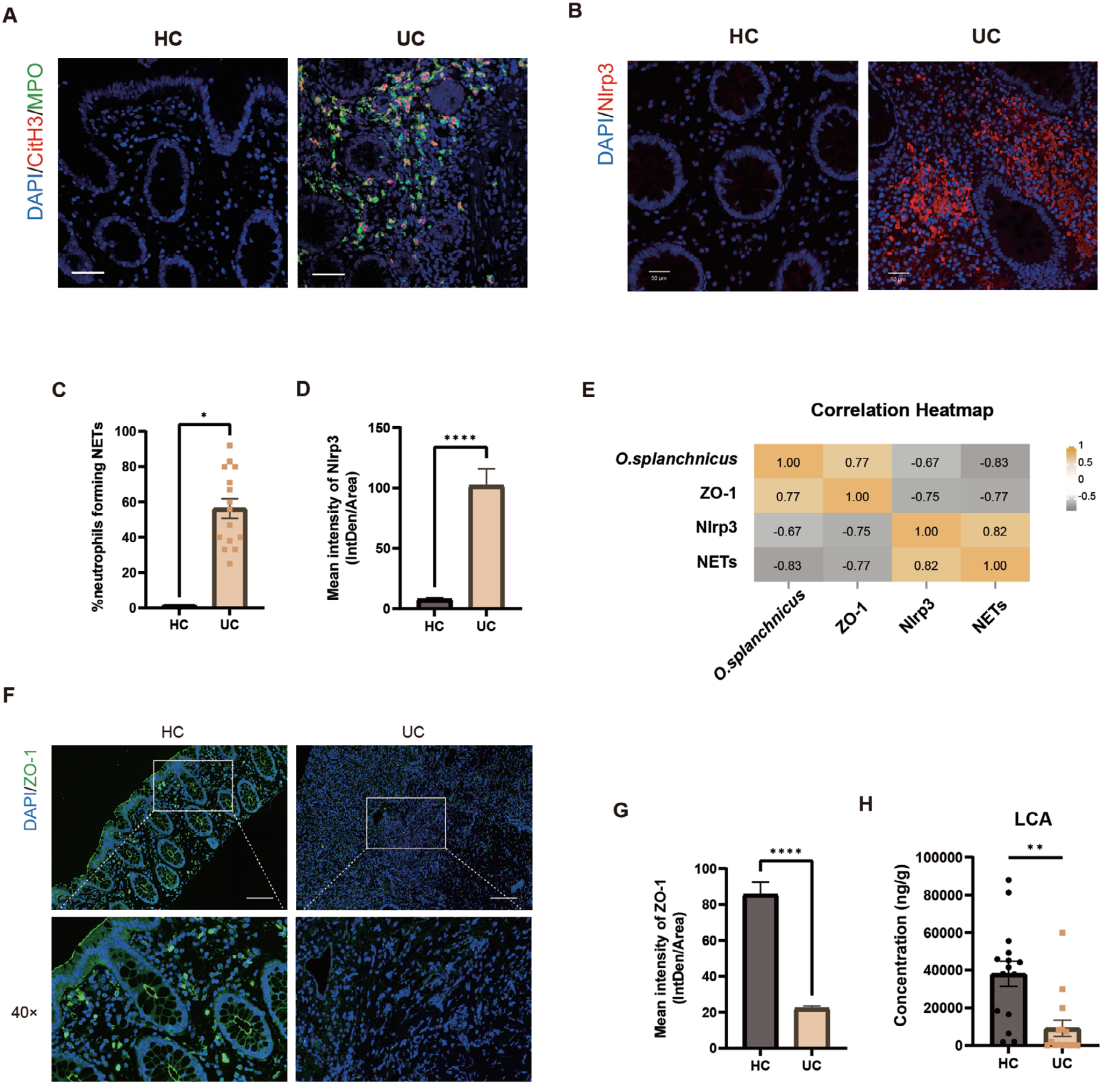
Decreased LCA concentration, increased NETs, and Nlrp3 expression were observed in patients with UC. A) Representative images of IF staining for CitH3 (red) and MPO (green) in patients with UC and healthy controls. B) Representative images of IF staining for Nlrp3 (red). DAPI was used to stain nuclei (blue). C) Statistical analysis of the percentage of NETs formation. Data are presented as mean ± SEM. ^*^, *P* < 0.05, as determined by unpaired Student's t test. D) Statistical analysis of Nlrp3 intensity in IF images. Data are presented as mean ± SEM. ^****^, *P* < 0.0001, as determined by unpaired Student's t test. E)The Spearman correlation heatmap (two‐tailed, *P* < 0.05) of *O. splanchnicus* abundance, ZO‐1 expression, Nlrp3 expression, and the percentage of NETs. F) Representative images of IF staining for the tight junction protein ZO‐1 (green) at 10× and 40× magnification, G), and statistical analysis of ZO‐1 intensity in IF images. H) Concentration of LCA in the feces of patients with UC and healthy control. Data are presented as mean ± SEM. ^*^, *P* < 0.05; ^**^, *P* < 0.01; ^****^, *P* < 0.0001, as determined by unpaired Student's t test.

To elucidate the clinical significance of the aforementioned findings, we next assessed the correlation between *O. splanchnicus* and NET formation in patients with UC. Compared with the HC group, the UC group exhibited increased NETs formation, as evidenced by enhanced colocalization of citrullinated histone 3 with MPO (**Figure** **8**A,C). In addition, we detected a pronounced increase in Nlrp3 expression (Figure 8B,D). A concomitant decrease in ZO‐1 expression was observed (Figure 8F,G), indicative of tight junction disruption and epithelial barrier impairment. A Spearman correlation heatmap was generated to explore the relationships between *O. splanchnicus* abundance, the percentage of NETs, Nlrp3 expression, and the tight junction protein ZO‐1 (Figure 8E). The results showed that *O. splanchnicus* abundance correlated positively with ZO‐1 expression (*r* = 0.77, *p* < 0.0001), whereas significant negative correlations were found with the percentage of NETs (*r* = −0.83, *p* < 0.0001) and Nlrp3 expression (*r* = −0.67, *p* < 0.0001). We also observed that LCA levels were significantly reduced in patients of UC (Figure 8H). Overall, these clinical data suggested a negative correlation between *O. splanchnicus* abundance and NETs formation in patients with UC, highlighting potential new therapeutic targets for UC.

## 3 Discussion

Damage to the intestinal barrier allows gut microbes to translocate, leading to excessive activation of the mucosal immune system, which drives the development of inflammation.^[^ ^25^ ^]^ The therapies for UC include 5‐ASA, corticosteroids, immunosuppressants, and biologics;^[^ ^26^ ^]^ however, patients with UC still face a high recurrence rate and the risk of adverse effects from the long‐term use of these drugs. In this study, we identified *Odoribacter splanchnicus* as a microbiota member capable of alleviating colonic inflammation and preserving barrier function in colitis. Our data revealed that these effects are mediated, at least in part, through suppression of NETs formation and microbiota‐dependent regulation of LCA. These findings extend previous reports linking microbiotal metabolites to host immune regulation, and provide direct evidence for a gut microbiota‐metabolite‐neutrophil axis in IBD.

Impaired intestinal mucosal barrier and dysbiosis are integral to the pathogenesis of IBD. Certain bacteria have been shown to interact with the immune system and exert protective effects on inflammation suppression, highlighting their strong potential for clinical translation. For instance, *C. butyricum*, a butyrate‐producing symbiont, played a protective role in IBD.^[^ ^27^ ^]^ Additionally, *F. prausnitizii* and its metabolites suppressed inflammation through the IL‐6/STAS3/IL‐17 axis and promoted FOXP3 expression.^[^ ^28^ ^]^ Studies also showed that the screening of IgA‐coated *O. splanchnicus* using a combined IgA coating index and machine learning proven to be a good predictor of the efficacy of FMT in patients with UC.^[^ ^15^ ^]^ Moreover, the response of IgA coating levels to the microbiota correlated with disease progression closely.^[^ ^29^ ^]^ In agreement with these findings, our study found that the relative abundance of *Odoribacter* and *O. splanchnicus* was significantly lower in our UC cohort. We also observed that increased IgA‐coated *O. splanchnicus* was present in patients with UC who responded to FMT treatment. Few studies have reported the association between *O. splanchnicus* and disease, but mono‐colonization with *O. splanchnicus* has been shown to suppress inflammation in colitis mice by inducing Foxp3^+^/RORγt^+^ Tregs.^[^ ^15^ ^]^ Xing et al.^[^ ^16^ ^]^ observed abundant enrichment of *O. splanchnicus* in *Tak1^∆M/∆M^* mice, with an evident induction of Th17 cell development following *O. splanchnicus* intervention, which protected against colitis and colorectal cancer. In line with these studies, our data showed that *O. splanchnicus* exerted a protective effect on inflammation suppression and intestinal barrier repair in both DSS‐induced colitis and *Il‐10^−/−^* spontaneous colitis mice models. Our results suggested that supplementation with *O. splanchnicus* might be a promising therapy for ameliorating UC progression and warrants further investigation. Several commensal microbes have been shown to modulate host immunity. For instance, *Bacteroides fragilis* modulated host immunity by producing structurally distinct glycolipids that regulate natural killer T (NKT) cell activity through CD1d‐dependent mechanisms.^[^ ^30^ ^]^ In addition, *Bacteroides fragilis* or its capsular polysaccharide A (PSA) exerted immunomodulatory effects by inducing IL‐10‐producing regulatory T cells via B cell‐dependent pathways, thereby protecting against excessive inflammation in both peripheral and CNS tissues. *Faecalibacterium prausnitzii* is a well‐known commensal bacterium with anti‐inflammatory properties. It has been shown to mitigate immune checkpoint inhibitor‐associated colitis by reducing myeloid cell‐driven intestinal inflammation and reshaping the gut microbiota, while also enhancing the efficacy of antitumor immunity.^[^ ^31^ ^]^ *Faecalibacterium prausnitzii* has also been shown to promote intestinal immune homeostasis by upregulating secretory IgA responses and reshaping the gut microbiota.^[^ ^32^ ^]^ In DSS‐induced colitis models, *F. prausnitzii* enhanced the expression of IgA‐related genes and reduced the abundance of pro‐inflammatory bacterial taxa, contributing to improved mucosal immunity and intestinal barrier integrity.^[^ ^32^ ^]^ These findings, along with our data on *O. splanchnicus*, highlighted the diverse mechanisms through which gut bacteria influence immune function. Our data found that *O. splanchnicus* can modulate the mucosal immune landscape, potentially providing a mechanism for alleviating inflammation and gut barrier injury. The recruitment and activation of neutrophils constitute the initial and pivotal stage of the inflammatory response.^[^ ^18^ ^]^ A high number of neutrophils was observed in the inflamed mucosa of patients with active UC, and their count may serve as a predictor of disease activity.^[^ ^33^ ^]^ Some studies have also shown gut microbiota plays a modulatory role in neutrophil homeostasis.^[^ ^34^ ^]^ Consistent with these findings, results from two colitis models with reduced neutrophil infiltration indicate that *O. splanchnicus* treatment modulates inflammatory and immune responses. The abrogation of this effect upon neutrophil depletion underscores neutrophils as indispensable mediators of *O. splanchnicus*‐induced protection.

Our data demonstrated that *O. splanchnicus* alleviated colonic inflammation and preserved barrier integrity by suppressing NETs formation, and that this protective effect was abolished in *Pad4* ^−/−^ mice. This finding underscored the requirement for PAD4‐dependent NETosis in mediating the anti‐inflammatory activity of *O. splanchnicus*. PAD4 catalyzes histone citrullination, a pivotal step in chromatin decondensation and DNA release.^[^ ^19^ ^]^ *Pad4* ^−/−^ mice failed to form NETs upon stimulation.^[^ ^35^ ^]^ In UC, excessive NETs accumulation amplified inflammatory signaling and drived IL‐1β and IL‐6 production, culminating in epithelial barrier disruption.^[^ ^36^ ^]^ NETs‐associated histones and proteases directly damaged epithelial cells and increased intestinal permeability,^[^ ^37^ ^]^ while elevated NETs levels correlated with epithelial injury in inflammatory conditions such as sepsis^[^ ^36^ ^]^ and ischemia‐reperfusion.^[^ ^38^ ^]^ Notably, *O. splanchnicus* intervention also reduced CD177 expression in specific neutrophil subclusters. Given that CD177⁺ neutrophils have been reported to display altered cytokine production and a greater capacity for NETs release,^[^ ^39^ ^]^ this downregulation may represent an additional mechanism by which *O. splanchnicus* limits NETs‐mediated mucosal damage. In our colitis model, *O. splanchnicus* treatment reduced both NETs formation and pro‐inflammatory cytokine expression. Moreover, upregulation of DNase expression following *O. splanchnicus* intervention suggested a potential contribution to NETs clearance, although further studies are needed to confirm its specific role in extracellular DNA degradation.^[^ ^40^ ^]^ Collectively, these results supported a model in which *O. splanchnicus* modulates neutrophil function and PAD4‐dependent NETosis to maintain mucosal integrity during colitis.

Gut microbiota‐host interactions are essential for maintaining immune homeostasis, in part through microbial metabolites such as BAs.^[^ ^41^ ^]^ Untargeted metabolomics in our study revealed enrichment of BA metabolism following *O. splanchnicus* intervention, suggesting this pathway as a potential mediator of its effects. Hepatic BA synthesis, regulated by cytochrome P450 enzymes through classical and alternative pathways,^[^ ^42^ ^]^ was enhanced in *O. splanchnicus*‐treated mice, as evidenced by upregulation of *Cyp7b1*, *Cyp27a1*, *Cyp2c70*, *Cyp7a1*, and *Cyp8b1*. In the presence of bile acid‐CoA synthetase (BACS) and bile acid‐CoA: amino acid N‐acyltransferase (BAAT), primary bile acids conjugate with taurine and glycine and are subsequently converted into conjugated bile acids, which are secreted into the gallbladder.^[^ ^43^ ^]^ Upon entering the bile duct, conjugated bile acids are excreted into the small intestine, promoting the absorption of lipids and vitamins.^[^ ^44^ ^]^ Bile acids are re‐absorbed by epithelial cells in the ileum and returned to the liver via the portal vein, a process known as enterohepatic circulation.^[^ ^45^ ^]^ Beyond synthesis, BA homeostasis is shaped by intestinal transport. ASBT mediates ileal BA reabsorption in enterohepatic circulation, while BSEP and OSTβ facilitate BA excretion.^[^ ^46^ ^]^ Consistent with this, *O. splanchnicus* treatment reduced ileal *Asbt* and *Ostβ* expression, indicating that it may influence bile acid homeostasis through decreased reabsorption in the enterohepatic circulation.

Alterations in BA profiles are associated with IBD, with enrichment of primary BAs and depletion of secondary BAs commonly observed in patients with CD and colectomy‐treated UC.^[^ ^47^ ^]^ Secondary BA enrichment and increased microbial diversity have been linked to improved therapeutic responses in IBD patients with receiving biologic therapy,^[^ ^48^ ^]^ whereas reduced abundance of BSH‐producing bacteria is frequently reported.^[^ ^49^ ^]^ In our study, targeted metabolomics showed that *O. splanchnicus* treatment decreased fecal conjugated and primary BAs while increasing unconjugated and secondary BAs. Notably, LCA levels were markedly elevated in line with untargeted metabolomics results. These changes reflected a shift toward a BA profile characteristic of a healthier gut environment. Moreover, LCA supplementation reproduced anti‐inflammatory effects observed with *O. splanchnicus*, raising the possibility that microbiota‐driven LCA upregulation contributes to its protective mechanism.

Microbial conversion of primary to secondary BAs requires sequential enzymatic reactions, starting with BSH‐mediated deconjugation and followed by structural modifications including 7α/β‐dehydroxylation catalyzed by bacterial enzymes.^[^ ^50^ ^]^ BSH‐containing taxa are primarily gram‐positive bacteria such as *Clostridium*, *Bifidobacterium*, *Lactobacillus*, and *Enterococcus*, with some gram‐negative members within Bacteroidetes.^[^ ^22^ ^]^ Secondary BAs, including DCA and LCA are mainly produced via 7α/β‐dehydroxylation mediated by bai genes, while alternative pathways such as epimerization and oxidation also contribute.^[^ ^23^, ^50^ ^]^ In our study, *O. splanchnicus* increased the abundance of BSH‐containing bacteria and taxa associated with the 7α/β‐dehydroxylation pathway, accompanied by elevated fecal LCA levels in mice. These findings suggested that *O. splanchnicus* may indirectly promote LCA production by modulating the gut microbial community.

To determine whether *O. splanchnicus* directly produces LCA, we performed in vitro co‐culture assays with CDCA as substrate and monocolonization experiments in GF mice. In both settings, *O. splanchnicus* did not significantly increase LCA levels, suggesting that LCA enrichment is unlikely to result solely from its intrinsic metabolic capacity. Interestingly, we detected LCA derivatives, including isoLCA and 12‐ketoLCA, in the conditioned medium of O. splanchnicus cultures following co‐incubation with CDCA. This observation suggested that the bacterium may directly catalyze downstream modifications of LCA to yield immunomodulatory derivatives. This is in line with reports that members of the *Odoribacteraceae* family can generate similar compounds, such as isoalloLCA,^[^ ^21^ ^]^ and that *Bacteroidetes* harbor biosynthetic gene clusters encoding enzymes including 5β‐reductase, 5α‐reductase, and 3β‐HSDH which involved in the transformation of 3‐oxoLCA to isoalloLCA.^[^ ^51^ ^]^ Consistent with a microbiota‐dependent mechanism, *O. splanchnicus* failed to alleviate colonic inflammation or restore barrier function in Abx‐treated mice, whereas FMT from *O. splanchnicus*‐treated donors restored both the anti‐inflammatory phenotype and the elevation of LCA and its derivatives. Collectively, these results indicated that *O. splanchnicus* confers protection primarily by reshaping the gut microbial community to enhance LCA production, which in turn modulated neutrophil‐mediated immune responses.

Bile acids, produced endogenously by both the host and the gut microbiota,^[^ ^52^ ^]^ exert profound effects on intestinal inflammation^[^ ^6^, ^47^ ^]^ and immune regulation.^[^ ^51^, ^53^ ^]^ Among them, microbiota‐transformed secondary bile acids have emerged as key modulators of immune homeostasis and IBD pathogenesis.^[^ ^54^ ^]^ Previous studies have largely focused on their actions on T cells and macrophages. For example, certain secondary bile acids and their derivatives inhibit Th17 cell differentiation by directly binding to RORγt,^[^ ^55^ ^]^ while isoalloLCA promotes Tregs development through conserved noncoding sequence 3.^[^ ^55^ ^]^ Conversely, elevated levels of DCA in colitis models drive macrophage polarization toward the pro‐inflammatory M1 phenotype.^[^ ^56^ ^]^ Genomic analyses have further identified biosynthetic gene clusters in *Bacteroidetes* responsible for converting 3‐oxoLCA to isoalloLCA, with both the metabolite and its biosynthetic genes found to be reduced in IBD patients.^[^ ^51^ ^]^ Together, these findings underscore the pivotal role of secondary bile acids in maintaining intestinal homeostasis. Building on this framework, our study demonstrated that secondary bile acids also modulate neutrophil‐driven inflammation. LCA supplementation significantly reduced neutrophil infiltration and NETosis both in vivo and in vitro. Collectively, the results showed that *O. splanchnicus* enhances LCA transformation in a gut microbiota‐dependent manner, thereby exerting immune‐modulatory effects on neutrophil infiltration and NETs formation in the colitis model. Notably, the conditioned medium of *O. splanchnicus* conferred partial anti‐inflammatory and barrier‐protective effects in vivo, yet it did not directly inhibit NET formation. This suggested that *O. splanchnicus* may also act through NET‐independent pathways and warrants further characterization.

Bile acid receptors play a role in regulating the effects of secondary bile acids on inflammation and mucosal immunity. FXR, VDR, and G protein‐coupled bile acid receptor 1 (GPBAR1) are among the most widely studied bile acid receptors. DCs deficient in FXR exhibit a transcriptional profile similar to that observed following isoDCA intervention,^[^ ^53b^ ^]^ suggesting that FXR interactions with isoDCA are correlated with the phenotype of isoDCA‐regulated DCs. In germ‐free mice mono‐colonized with *Bacteroides* strains, Treg differentiation is induced via VDR and FXR.^[^ ^53a^ ^]^ Some studies have also indicated that FXR and its target gene *FGF19* play a protective role in colitis.^[^ ^57^ ^]^ In addition, some studies demonstrated that secondary bile acids can promote the regeneration of intestinal organoids by activating TGR5 in intestinal stem cells.^[^ ^58^ ^]^ The TGR5 analog INT‐777 can similarly promote stem cell regeneration.^[^ ^58^ ^]^ The protective effect of secondary bile acids was absent in mice with immune cell‐specific deletion of TGR5.^[^ ^59^ ^]^ In alignment with these findings, our results indicated that *O. splanchnicus* intervention upregulates *Tgr5* expression, further suggesting that its effects may be linked to the regulation of bile acid metabolism. However, changes in *Fxr* expression were not significant following *O. splanchnicus* intervention, which may be due to differences in sampling time points and locations.

Another novel finding is that LCA, elevated after *O. splanchnicus* intervention, directly inhibited the NLRP3 signaling pathway. This inhibition contributed to reduced NETosis and alleviated colitis. Several studies have reported interactions between bile acids and the inflammasome.^[^ ^24^, ^60^ ^]^ LCA promoted the phosphorylation and ubiquitination of macrophage NLRP3 through activation of TGR5, thereby inhibiting NLRP3‐mediated inflammation.^[^ ^24^ ^]^ Moreover, the modulation of bile acid receptors to inhibit NLRP3 activation has been shown to influence inflammation associated with nonalcoholic hepatitis.^[^ ^60^ ^]^ Consistent with these findings, we observed reduced NLRP3 expression and lower cleaved GSDMD activation in mice after bile acid intervention, which accounts for the LCA‐induced inhibition of NETs. Some studies have found that neutrophils and macrophages play a role in autoimmune diseases through interactions with the inflammasome.^[^ ^61^ ^]^ Increased NETosis promoted macrophage inflammasome activation, thereby contributing to disease progression and activity in patients with lupus.^[^ ^61^ ^]^ In this study, we found that NLRP3 expression in neutrophils was reduced following *O. splanchnicus* intervention. Additionally, the anti‐inflammatory effects of *O. splanchnicus* and LCA were abrogated in mice lacking Nlrp3 expression. Beyond the direct effects of the NLRP3‐GSDMD axis on NETosis modulated by LCA, NLRP3 signaling also contributes to *O. splanchnicus*‐induced inhibition of inflammation and barrier repair in colitis. Our findings suggested that LCA, modulated by *O. splanchnicus*, significantly inhibited the infiltration of neutrophils and NETosis by decreasing the expression of the NLRP3‐GSDMD axis in colitis mice.

Notably, our finding of a depleted *O. splanchnicus* in patients with UC and an increased abundance of IgA‐coated *O. splanchnicus* in patients with UC responding to FMT therapy is consistent with previous studies,^[^ ^17a^ ^]^ which demonstrated that *O. splanchnicus* may serve as a potential probiotic to predict treatment efficacy and better regulate immune homeostasis. In two different models, DSS‐induced acute colitis in mice and the Il‐10^−/−^ spontaneously occurring chronic IBD model, we demonstrated that *O. splanchnicus* intervention significantly protects against inflammation and aids in gut barrier repair. In conclusion, the transformation of secondary bile acids by *O. splanchnicus* modulated the balance between the formation and degradation of NETs, thereby protecting against intestinal inflammation and gut barrier injury. Although our study centered on the LCA‐NETs axis, *O. splanchnicus* treatment also induced transcriptional alterations in other immune cell populations, including B cells, T cells, and macrophages. Combined with the in vivo anti‐inflammatory activity of its conditioned medium and the detection of potentially protective metabolites in the bacterial supernatant, these findings point to broader immunomodulatory effects beyond neutrophils, potentially mediated by *O. splanchnicus*‐derived metabolites. Further investigation is warranted to elucidate these additional pathways and their translational relevance. Together, this highlighted the mechanisms through which gut microbiota‐host interactions maintain homeostasis via bile acid‐mediated mucosal immune modulation. Hence, *O. splanchnicus* might serve as a probiotic therapy for dysbiosis‐driven diseases, helping restore intestinal homeostasis.

## 4 Experimental Section

### Clinical Specimens

Cohort 1 consisted of 15 patients with UC and 15 healthy controls, enrolled between 2020 and 2021. A total of 15 paired stool samples were used to examine the abundance of *O. splanchnicus* and to validate bile acid concentrations via LC‐MS. Additionally, eight formalin‐fixed, paraffin‐embedded tissue samples were included in Cohort 1. These sections were used to visualize the colocalization of citH3‐MPO and citH3‐NE, as well as the expression of ZO‐1 and NLRP3 in UC tissues and control non‐inflammatory tissues. Cohort 2 comprised 14 patients with UC and 195 healthy donors who underwent FMT between 2018 and 2020. We re‐analyzed the 16S rRNA data to primarily validate the relative abundance of *O. splanchnicus* in UC. Cohort 3 included 16 patients with UC who received FMT treatment between 2020 and 2022. We reanalyzed the IgA‐seq data to explore the abundance of IgA‐coated *O. splanchnicus* before and after FMT treatment. The clinical retrospective studies and analyses were approved by the Ethics Committee of Guangzhou First People's Hospital (South China University of Technology, School of Medicine; S‐2023‐016‐01). All participants provided informed consent.

### Bacterial Culture

*O. splanchnicus* (ATCC‐29572) was purchased from the American Type Culture Collection (ATCC). The bacteria were incubated for 48 h at 37 °C under anaerobic conditions (LABIOPHY, Dalian, China) in Gifu anaerobic medium (GAM) broth, supplemented with 1 µg/mL of vitamin K1 and 5 µg mL^−1^ of hemin. To maintain its activity, we subcultured *O. splanchnicus* on Columbia Blood Agar for 48 h, and a single colony was subsequently selected for expansion in GAM broth at 37 °C under anaerobic conditions.

### Treatmentof O. splanchnicus

For bacterial treatment, *O. splanchnicus* in the logarithmic growth phase was harvested and centrifuged at 5000 ×*g* for 15 min at 4 °C. The bacterial pellets were washed twice with 10 mL of cold PBS buffer and resuspended to a concentration of 10^8^ CFU/mL (OD_600_ = 1) in 200 µL. Mice were subjected to daily gavage intervention for 1 week or 10 days, with PBS used as the control. To prepare heat‐inactivated bacteria, we heated *O. splanchnicus* at 100 °C for 30 min.

### *Teatment of* O. splanchnicus Supernatant

For the preparation of the conditional medium, *O. splanchnicus* at the logarithmic growth stage was harvested and centrifuged at 5000 × *g* for 15 min at 4 °C. The supernatant was filtered through a 0.22 µm filter to remove bacteria, yielding the conditional medium. A daily oral dose of 200 µL of the medium was administered for either 1 week or 10 days during the experiments. GAM was used as a placebo.

For the protease K treatment, the conditional medium of *O. splanchnicus* was supplemented with 100 µg mL^−1^ of protease K and incubated at 50 °C for 1 h, followed by a 5 min incubation at 100 °C. To separate different fractions of the *O. splanchnicus* conditional medium, we obtained the <3 kDa fraction by centrifuging the conditional medium through an Amicon Ultra Centrifugal Filter with a 3000 NMWL membrane (Millipore, USA), following the manufacturer's protocol. GAM was prepared in the same manner as the conditional medium and used as the vehicle.

### *Teatment of* LCA

For the LCA intervention, 50 mg kg^−1^ of LCA was administered orally on a daily basis for either 1 week or 10 days during the experiments. The solvent was used as the vehicle.

### Experimental Animals

The Experimental Animal Ethic Committee of South China University of Technology approved animal experiments (82470579). Wild type C57BL/6 mice were purchased from the Guangdong Province Animal Center (Guangzhou, China) and were housed under specific pathogen‐free (SPF) conditions. *Il‐10^−/−^*, *Pad4^−/−^*, and *Nlrp3^−/−^* mice were purchased from the Shanghai Model Organisms Center, Inc. (Shanghai, China) and were also housed under SPF conditions. Germ‐free C57BL/6 mice were purchased from Gnotobio (Shenzhen, China) and maintained under SPF conditions. Mice aged 6 to 10 weeks were used for the experiments and were randomly assigned to different groups, with a maximum of six mice per cage. All animals were housed at a room temperature of 25 °C with a 12 h light/dark cycle to maintain their circadian rhythm. The animal experiments were approved by the Institutional Animal Care and Use Committee of the School of Medicine, South China University of Technology.

For the DSS‐induced SPF and *Pad4^−/−^* mouse model, 7‐week‐old male C57 wild type mice were acclimatized for 1 week, followed by administration of 2.5% or 3% DSS in drinking water for 10 days or 7 days, respectively. *O. splanchnicus* was administered by gavage once daily throughout the entire modeling period. The following parameters were recorded: weight loss, perianal condition, stool properties, and the presence of occult blood in the stool. For the DAI analysis, weight loss, stool properties, and occult blood in the stool were observed and used to calculate DAI scores, following a previously established protocol.^[^ ^62^ ^]^ For the *Il‐10^−/−^* mouse model, 9‐week‐old male *Il‐10^−/−^* mice were housed for 1 week prior to the study for acclimatization. *O. splanchnicus* was administered by oral gavage three times a week for 13 weeks. The following parameters were recorded: weight loss, perianal condition, and stool properties. For the DSS‐induced germ‐free mouse model, 8‐week‐old male germ‐free mice were housed in a specialized facility with a sterile environment. To promote better colonization by *O. splanchnicus*, we treated the mice daily with *O. splanchnicus* for 2 weeks. In the final week of the study, 2% DSS was provided ad libitum to establish a colitis model. The following parameters were observed and recorded: weight loss, stool properties, and the presence of occult blood in the stool. For the antibiotic cocktail mouse model, ampicillin (1 g L^−1^), neomycin (1 g L^−1^), metronidazole (1 g L^−1^), and vancomycin (0.5 g L^−1^) were added to the drinking water of mice ad libitum for 4 weeks to eliminate most intestinal bacteria. For FMT treatment, 1 g of freshly collected mouse feces was homogenized in 10 mL of PBS. The fecal homogenate was passed through a 100 µm filter, and the supernatant was collected. After 4 weeks of antibiotic cocktail pretreatment, each mouse was administered 200 µL of fecal supernatant by gavage once daily for 1 week, while consuming 3% DSS in drinking water ad libitum. The following parameters were observed and recorded: weight loss, stool properties, and the presence of occult blood in the stool. For the *Nlrp3^−/−^* mouse model, 9‐week‐old male *Nlrp3^−/−^* mice and wild type controls were acclimatized and fed for 1 week. The mice were given 3% DSS ad libitum for 1 week, while *O. splanchnicus* (10^8^ CFU/mL) or LCA (50 mg/kg) were administered by gavage once daily for 1 week respectively. For neutrophils depletion experiments, acute colitis was induced by providing 3% DSS in drinking water for 7 days. Mice in the *O. splanchnicus* (10^8^ CFU/mL) group received daily oral gavage during the DSS induction period. In the neutrophil depletion groups, anti‐mouse Ly6G antibody (BioXcell, 200 µg/mouse, intraperitoneally on days −1, 1, 3, and 5) was administered intraperitoneally on days −1, 1, 3, and 5 relative to DSS induction.

### Isolation and Stimulation of Bone Marrow Neutrophils

After euthanasia, femurs and tibias of mice were harvested and flushed with PBS to collect bone marrow cells as the previous protocol.^[^ ^63^ ^]^ Briefly, neutrophils were isolated by Percoll density gradient centrifugation. Red blood cells (RBCs) were eliminated by incubating with RBC lysis buffer. Purified neutrophils were seeded at 4.5 × 10⁶ cells per well and incubated for 1 h. The PMA group was treated with 400 nM phorbol 12‐myristate 13‐acetate (PMA; MCE, HY‐18739) for 12 h. The LCA group was stimulated with PMA in combination with either 5 µM or 10 µM LCA. The CM group received PMA together with *O. splanchnicus* bacterial conditioned medium at final concentrations of 5%, 10%, or 20% (v/v). Nuclear staining was performed using Hoechst 33342 (Beyotime, C1029). NET formation was assessed using a Zeiss LSM 900 confocal microscope (ZEN 3.10 software) under strictly aseptic conditions.

For detecting the NETs formation by immunofluorescence staining, Cells were fixed with 4% paraformaldehyde at room temperature for 30 min, permeabilized with 0.1% Triton X‐100 in PBS for 3 min, and then blocked with 2% bovine serum albumin (BSA) in PBS for 30 min. Multi‐immunofluorescence staining was performed following the manufacturer's protocol using the TSA staining kit (Servicebio, G1226‐100T). The primary antibodies including anti‐MPO (Abcam, ab208670), anti‐H3Cit (Abcam, ab5103). Fluorescence signals were visualized using a Zeiss LSM 900 confocal microscope equipped with ZEN 3.10 software.

### O. splanchnicus detection

Fecal DNA was extracted using the QIAamp PowerFecal DNA Kit (Qiagen, Germany) according to the manufacturer's instructions. qPCR was then employed for the relative quantification of *O. splanchnicus* in feces. The abundance of *O. splanchnicus* was determined by calculating the ΔCT value, defined as the difference between the Ct value for *O. splanchnicus* and the Ct value for the 16S gene (16S).^[^ ^64^ ^]^ The primers for *O. splanchnicus* and the universal 16S gene are listed in Table S1.

### High Throughput 16S rRNA‐Sequencing

The composition of the gut microbiota was assessed using high‐throughput 16S rRNA sequencing. Fecal DNA was extracted using the HiPure Soil DNA Kit (Magen, Guangzhou, China) according to the manufacturer's instructions. DNA concentration and quality were determined using a Nanodrop spectrophotometer. Primers targeting the bacterial 16S ribosomal RNA gene (341F: 5′‐CCTACGGGGNGGCWGCAG‐3′ and 806R: 5′‐GGACTACHVGGGGTATCTAAT‐3′) were used to amplify the V3‐V4 region via PCR. After purification and library construction, sequencing was performed on the Novaseq 6000 platform (Illumina, CA, USA). Sequencing data were processed and further analyzed using the Omicsmart platform (Gene Denovo co., Guangzhou, China).

### SEM

The morphology of the tissue surface of *O. splanchnicus* was observed and analyzed using a scanning electron microscope (LEO 1530vp, Germany).

### HE Staining

A 0.5 cm segment of the terminal rectum was collected from colitis model mice on day 7 or day 10. The tissues were fixed in 4% paraformaldehyde for 24 h. After fixation, the tissues underwent a series of dehydration, clearing, and paraffin embedding processes, followed by sectioning and staining according to standard protocols.^[^ ^65^ ^]^ Image analysis was performed using a Nikon optical microscope after staining. For the DSS‐induced colitis mouse model, the histological score was calculated based on the extent of mucosal damage and immune infiltration.^[^ ^66^ ^]^ Two pathologists independently confirmed the histological score to ensure a more accurate evaluation.

### RNA Extraction and Real‐Time qPCR

Total RNA was extracted from liver, ileum, and colon tissues preserved in RNAlater using TRIzol reagent (Thermo Fisher Scientific, USA). Reverse transcription of RNA to synthesize complementary DNA was performed using the PrimeScript RT Reagent Kit with gDNA Eraser (RR047A, Takara), following Nanodrop assessment of RNA concentration and quality. Real‐time qPCR was conducted using TB Green Premix Ex Taq II (Tli RNaseH Plus) (RR820A, Takara). Ct values were detected using an Applied Biosystems QuantStudio 5 (Thermo Fisher Scientific). Relative mRNA expression was calculated using the 2^−∆∆Ct^ method with β‐actin as the internal reference.^[^ ^64^ ^]^ Specific primer sequences are provided in Table S1.

### IF for Paraffin‐Embedded Sections

For IF, the slides were stained using previously described methods.^[^ ^67^ ^]^ Briefly, the sections were incubated at 60 °C for 2 h, followed by a series of deparaffinization and rehydration steps. For antigen repair, the sections were incubated in 1× EDTA repair solution at 100 °C for 15 min. The sections were allowed to cool naturally and then washed three times with PBS. After blocking with 1% BSA, incubation was performed with the following primary antibodies overnight at 4 °C: anti‐Zo‐1 (1:200; Abcam, ab216880), anti‐Occludin (1:2000; Proteintech, 27260‐1‐AP), anti‐Claudin1 (1:200; Proteintech, 28674‐1‐AP), anti‐Muc2 (1:200; Novus Biologicals, NBP1‐31231), anti‐Pad4 (1:200; Abcam, ab96758), and anti‐Nlrp3 (1:50; Novus Biologicals, NBP2‐12446), respectively. After incubation with secondary antibodies conjugated to Alexa Fluor 488 (1:500; Abcam, ab150077) or Alexa Fluor 555 (1:200; Abcam, ab150078) for 50 min at room temperature, the sections were stained with DAPI (C1005, Beyotime) to visualize cell nuclei. Images were captured using a confocal microscope (Leica) and a scanner (Akoya Biosciences). Each section was analyzed at 40× magnification in at least five fields of view, and subsequent statistical analysis was performed using ImageJ software 1.50i.

For multiplex IF, the slides were stained using the tyramide signal amplification assay kit (Servicebio, China) according to the manufacturer's instructions. After deparaffinization and hydration, the sections were incubated in 1× EDTA antigen retrieval solution for 15 min at 100 °C to repair the antigens. After natural cooling, a PAP pen was used to circle the tissue area. The slides were blocked with 1% BSA for 30 min at room temperature and incubated with the first primary antibody overnight at 4 °C. The slides were then stained with horseradish peroxidase (HRP)‐conjugated secondary antibody (1:200; Abcam, ab133357) for 50 min at room temperature, followed by staining with iF555‐Tyramide dye for 10 min at room temperature. This procedure was repeated for the second protein using the second primary antibody and iF488‐Tyramide dye or FITC‐conjugated anti‐Gr‐1 (1:100, Cell Signaling Technology, 88876S). Nuclei were stained with DAPI, and the slides were mounted with an antifade mounting medium, covered with a coverslip, and evaluated using a confocal fluorescence microscope (Leica) and scanner (Akoya Biosciences). Each section was analyzed at 40 × magnification in at least five fields of view, and the data were used for subsequent statistical analysis in ImageJ. The primary antibodies used included anti‐CitH3 (1:1000, Abcam, ab281584), anti‐MPO (1:500, Abcam, ab208670), anti‐Ne (1:200, Invitrogen, PA5‐87158), anti‐Nlrp3 (1:50; Novus Biologicals, NBP2‐12446), and anti‐Gr‐1 (1:100, Cell Signaling Technology, 88876S).

For the quantification of NET‐forming cells, at least three mice were analyzed for each experimental group following the established protocol.^[^ ^68^ ^]^ One representative colonic section was selected from each mouse, and immunofluorescence images were acquired at 40× magnification. Within each image, five randomly chosen fields of view were manually assessed by two dependent observers. Neutrophils were identified based on positive staining for MPO. NET‐forming neutrophils were defined by the extracellular localization of citrullinated histone H3 co‐stained with MPO. The percentage of NET‐forming neutrophils was calculated as (NET⁺/total neutrophils) × 100.

### Immunohistochemistry

To examine the expression of intestinal barrier‐related proteins, we collected the colon tissue from mice subjected to different treatments and fixed them as described previously.^[^ ^69^ ^]^ After embedding and sectioning, the tissue sections were blocked and incubated overnight at 4 °C with the following primary antibodies: anti‐Zo‐1 (1:500; Abcam, ab276131), anti‐Occludin (1:500; Proteintech, 27260‐1‐AP), anti‐Claudin1 (1:500; Proteintech, 28674‐1‐AP), and anti‐Muc2 (1:100; Novus Biologicals, NBP1‐31231). After washing, the sections were stained with an HRP‐conjugated secondary antibody (1:200; Abcam, ab6721) and counterstained with hematoxylin. To evaluate the IHC scores, we calculated the sum of the positive staining area and staining intensity as the reference.^[^ ^70^ ^]^

### AB‐PAS Staining

Colonic tissue from mice was collected and fixed in 4% paraformaldehyde overnight at 4 °C. After fixation, the tissue was embedded in paraffin and sectioned into 4 µm slices. The deparaffinized and rehydrated sections were stained using an AB‐PAS Staining Kit (G1049, Servicebio) according to the manufacturer's instructions. For imaging and analysis, stained sections were photographed and evaluated using a scanner (Akoya Biosciences). At least five random fields per slide were examined to calculate the number of goblet cells.

### Single‐Cell RNA Sequencing

Colonic tissues were isolated from DSS‐induced mice, with or without *O. splanchnicus* intervention, and subsequently minced and digested into a single‐cell suspension according to a previously established protocol[4]. Then, trypan blue was used to stain the obtained single cells. The counting of live cells was conducted using the Countess II Automated Cell Counter. Samples with ≥90% viable cells were selected for subsequent sequencing. Single‐cell capture and standard library preparation were conducted by Gene Denovo Co. (Guangzhou, China). Briefly, 500–1000 cells µL^−1^ of each sample were mixed with Single Cell 3′ gel beads and enzymes. This mixture was encapsulated in oil surfactant droplets on a Single Cell A Chip (10X Genomics, Chromium), forming Gel Beads‐In‐Emulsions (GEMs), which were then captured by the Chromium Single Cell Controller (10X Genomics). Real‐time RNA transcript sequencing based on unique cell barcodes was performed, followed by PCR amplification to generate cDNA for constructing the standard library. The single‐cell library was sequenced on the Illumina NovaSeq X Plus platform (Illumina).

To analyze the 10X Genomics scRNA‐seq data, we converted raw BCL files to FASTQ files using the 10X Genomics Cell Ranger software. The data were then aligned to the reference genome, with low‐quality reads filtered out. Gene expression quantification was performed using the EmptyDrops method. Downstream analysis, including cell clustering, was conducted using Seurat (version 3.1.1), and differentially expressed genes were identified. Additional analyses were performed on the Omicsmart platform (Gene Denovo Co., Guangzhou, China).

### Flow Cytometry

Single‐cell suspensions were prepared from the colonic tissue of mice according to a previously established protocol[4]. Briefly, freshly collected colon tissue was cut into small pieces and incubated in a digestion buffer containing DNase I (0.5 mg/mL, Roche) and collagenase IV (0.5 mg mL^−1^, Roche). After grinding, the tissue was filtered through a 70 µm filter to obtain a single‐cell suspension, which was then centrifuged. Dead cells were excluded using the Zombie Aqua Fixable Viability Kit (BioLegend). The cells were then stained with PerCP anti‐mouse CD45 (BioLegend), APC anti‐mouse CD11b (BioLegend), and PE/Cy7 anti‐mouse Gr‐1 (BioLegend) to analyze neutrophil changes. The detection of events was conducted using a BD LSRFortessa flow cytometer (BD Biosciences), and FlowJo software was used to analyze and quantify the percentage of immune cells.

### Bulk RNA Sequencing

The mRNA was extracted from colonic tissue using the CTAB method, followed by RNA sequencing according to a previously established protocol[4]. Briefly, the isolated mRNA was assessed for both quantity and quality. Enrichment and purification of the mRNA were then conducted. Next, the fragmented mRNA was used to synthesize cDNA, which was sequenced using the Illumina NovaSeq X Plus platform (Gene Denovo, Guangzhou, China). Downstream statistical analyses were conducted using R software and the Omicsmart platform (Gene Denovo, Guangzhou, China). R packages, including HISAT2, edgeR, and DESeq2, were used.

### Western blot analysis

Total protein was extracted from colon tissue and quantified using a BCA kit (Thermo Fisher Scientific, Waltham, MA, USA). SDS‐PAGE gels (10%–12%) were used to separate the total proteins from different groups. The separated proteins were then transferred to PVDF membranes (Millipore, Burlington, MA, USA). To block nonspecific binding, we incubated the membranes with 5% BSA non‐fat milk for 1 h at room temperature. After blocking, the membranes were incubated with primary antibodies overnight at 4 °C. The membranes were then incubated with HRP‐conjugated secondary antibody for 1 h at room temperature. Protein bands were visualized using SuperSignal West Pico PLUS Chemiluminescent Substrate (Thermo Fisher Scientific, Waltham, MA, USA), and images were captured using Image Lab (Bio‐Rad). The gray intensity of the bands was analyzed using ImageJ software. The details of the primary antibodies used were as follows: rabbit anti‐Pad4 monoclonal antibody (1:1000; Abcam, ab214810), rabbit anti‐Mpo monoclonal antibody (1:1000, ab208670), rabbit anti‐Ne polyclonal antibody (1:2000, PA5‐87158), rabbit anti‐CitH3 polyclonal antibody (1:1000, ab5103), rabbit anti‐DNase polyclonal antibody (1:1000, ab224617), rabbit anti‐β‐actin monoclonal antibody (4947S), rabbit anti‐Nlrp3 polyclonal antibody (1:500, NBP2‐12446), rabbit anti‐Asc monoclonal antibody (1:1000, 67824T), rabbit anti‐Il‐18 monoclonal antibody (1:1000, 57068S), and rabbit anti‐Gsdmd monoclonal antibody (1:1000, ab219800). The secondary antibodies used were: anti‐rabbit HRP‐linked secondary antibody (1:5000, 7074S) and anti‐mouse HRP‐linked secondary antibody (1:5000, 7076S).

### ELISA

The concentration of CitH3 in the plasma of mice was measured using an ELISA kit (501620, Cayman), while double‐strain DNA was quantified using the Quant‐iT PicoGreen dsDNA kit (P11496, Invitrogen), following the manufacturer's instructions.

### Untargeted Metabolomics and Targeted Bile Acid Metabolomics Analysis

For untargeted metabolomics sequencing, stool samples from mice were resuspended in methanol and vortexed thoroughly. After centrifugation and incubation, the supernatant was analyzed using an LC‐MS/MS system with an AB SCIEX Triple TOF 6600 (Gene Denovo Co., Guangzhou, China). The data were processed through quality control, cluster analysis, multivariate statistical analysis (including principal component analysis, partial least squares discriminant analysis, and orthogonal partial least squares discriminant analysis), and differential metabolite analysis. Differential metabolites were identified based on a VIP >1 and *P* < 0.05. For targeted metabolomic analysis of bile acids, stool samples and conditioned medium from OS were collected and prepared for detection using an LC‐MS system (Waters Acquity UPLC/AB SCIEX 5500 QQQ‐MS). Briefly, 50 mg of stool samples or 1 mL of freeze‐dried liquid samples were extracted by adding 600 µL cold methanol. The mixture was incubated for 30 min at 4 °C, then centrifuged at 12000 rpm for 10 min. The supernatant was collected and evaporated to dryness. Next, 200 µL of methanol containing 43 standard bile acid compounds was added to dissolve the powder. After centrifugation at 12000 rpm for 15 min, the supernatant from each sample was detected and quantified using the platform.

### In vitro Analysis of LCA Transformation by O. Splanchnicus

The logarithmic growth stage of *O. splanchnicus* (OD = 0.1) was inoculated into a medium containing bile acid substrates: CDCA (HY‐76847, MCE), CA (HY‐N0324, MCE), LCA (L6250, Sigma), and UDCA (HY‐13771, MCE). The concentration of bile acid in the *O. splanchnicus* supernatant was measured after 72 h of incubation at 37 °C in an anaerobic workstation using an LC‐MS system (Waters Acquity UPLC/AB SCIEX 5500 QQQ‐MS). Simultaneously, OD_600_ was measured with a Microplate Reader (Bio‐Rad) to evaluate the growth of *O. splanchnicus*.

### Statistical Analyses

The statistical analysis was performed using GraphPad Prism (9.4.1) and SPSS 20.0 (IBM, USA). Data are presented as the mean ± standard error of the mean (SEM). To compare differences between two groups, we applied the Student's t test to normally distributed data, while the Mann‐Whitney U test was used for non‐normally distributed data. For comparisons across multiple groups, one‐way ANOVA was used for normally distributed data, and the nonparametric Mann‐Whitney U test was applied to non‐normally distributed data. A *P*‐value of less than 0.05 was considered statistically significant.

## Conflict of Interest

The authors declare no conflict of interest.

## Author Contributions

JX designed, performed experiments, wrote the manuscript, analyzed the data, collected the samples, performed the statistical analysis. JHL conceived, analyzed the data, performed experiments, revised the manuscript. XG conceived, analyzed the data, collected the samples. CH performed the statistical analysis. YP recorded and analyzed the data, revised the manuscript. HMX recorded and analyzed the data, provided the funding support. YFL collected the samples. JKX supported on bulk RNA sequencing analysis. JXH supported on 16S rRNA sequencing analysis. YTL performed the experiments, recorded the data. YQN supervised the study, revised the manuscript, provide the funding support. YLZ provided the funding support, revised the manuscript, supervised the study. JX, JHL contributed equally to this work.

## Ethics Approval and Consent to Participate

The Experimental Animal Ethic Committee of South China University of Technology approved animal experiments (82470579). The clinical retrospective studies and analyses were approved by the Ethics Committee of Guangzhou First People's Hospital (South China University of Technology, School of Medicine; S‐2023‐016‐01). The written informed consent was obtained from the individual.

## Supporting information



Supporting Information

## Data Availability

All data are included in the article and supplementary materials. The 16S rRNA sequencing data of cecal contents was available in sequence read archive (SRA) database (The BioProject accession number: PRJNA1261584).
