# Supplementary material for: Secondary Bile Acids Modified by Odoribacter Splanchnicus Alleviate Colitis by Suppressing Neutrophil Extracellular Trap Formation
Source: Adv Sci (Weinh). 2025 Sep 24;12(46):e09073. doi: 10.1002/advs.202509073 (PMC12697870; doi:10.1002/advs.202509073)
Supplement: Supplementary file 1 — Supporting Information [file ADVS-12-e09073-s001.pdf]

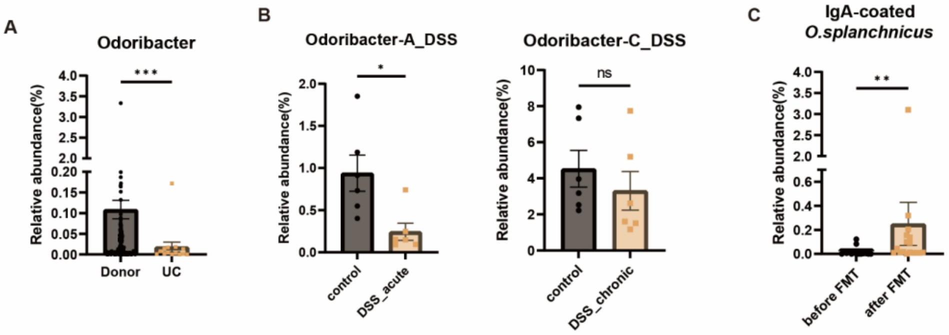

Supplementary Figure 1. (A) Comparison of the genus of *O. splanchnicus* in fecal samples from patients with newly diagnosed UC and healthy controls. Data are presented as mean ± SEM. **, *P* < 0.01; ***, *P* < 0.001, as determined by the Mann–Whitney unpaired nonparametric test. (B) Comparison of Odoribacter abundance in the feces of mice with acute or chronic colitis models and the control group. Data are presented as mean ± SEM. *, *P* < 0.05, as determined by the Mann–Whitney unpaired nonparametric test. (C) The abundance of IgA-coated *O. splanchnicus* was increased in patients with UC following FMT. Data are presented as mean ± SEM. **, *P* < 0.01, as determined by the Mann–Whitney unpaired nonparametric test.

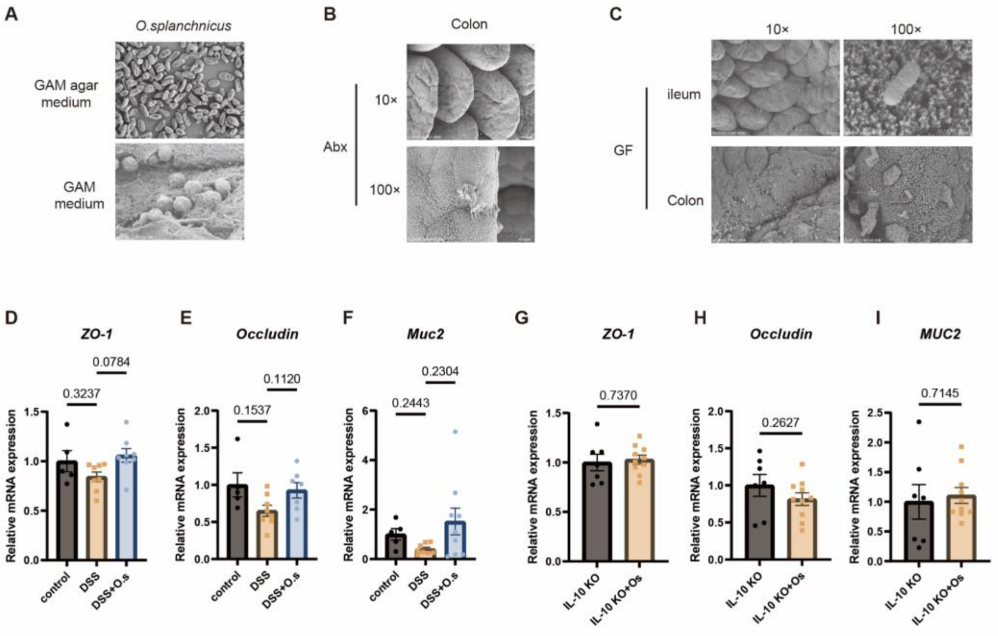

Supplementary Figure 2. (A) SEM revealed the morphology and presence of *O. splanchnicus* on the agar medium, broth medium, (B) intestinal surface of Abx-pretreated mice, (C) and germ-free mice. (D–F) The mRNA expression of gut barrier-related genes *Zo-1*, *Occludin*, and *Muc2* in DSS-induced colitis mice and (G–I) *Il-10*$^{-/-}$ mice.

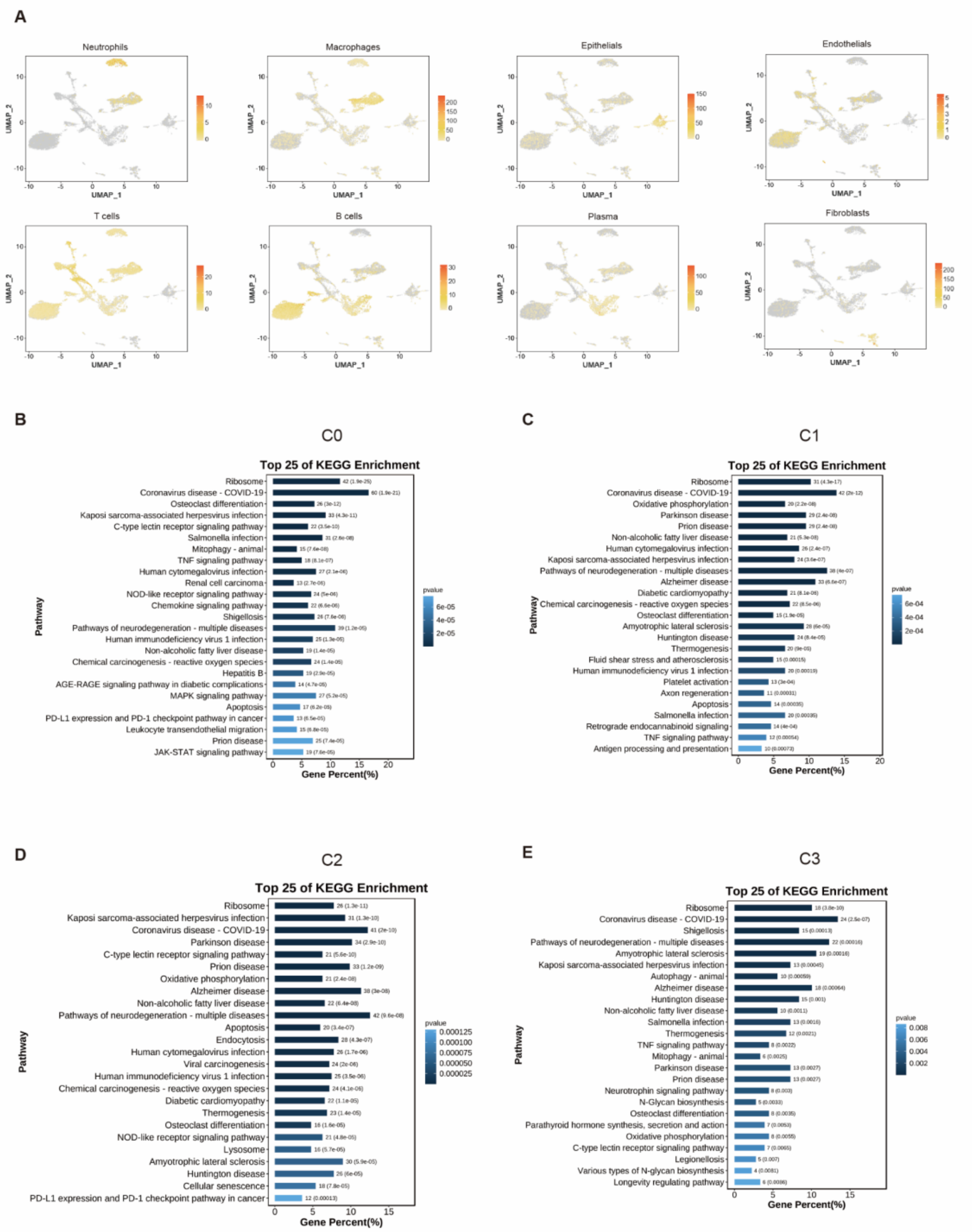

Supplementary Figure 3. (A) UMAP plot showing the clusters and marker genes of eight cell types following *O. splanchnicus* intervention. (B–E) The top 25 KEGG pathways enriched in neutrophil subpopulations after *O. splanchnicus* intervention. The subclusters include (B) Cluster 0, (C) Cluster 1, (D) Cluster 2, and (E) Cluster 3.

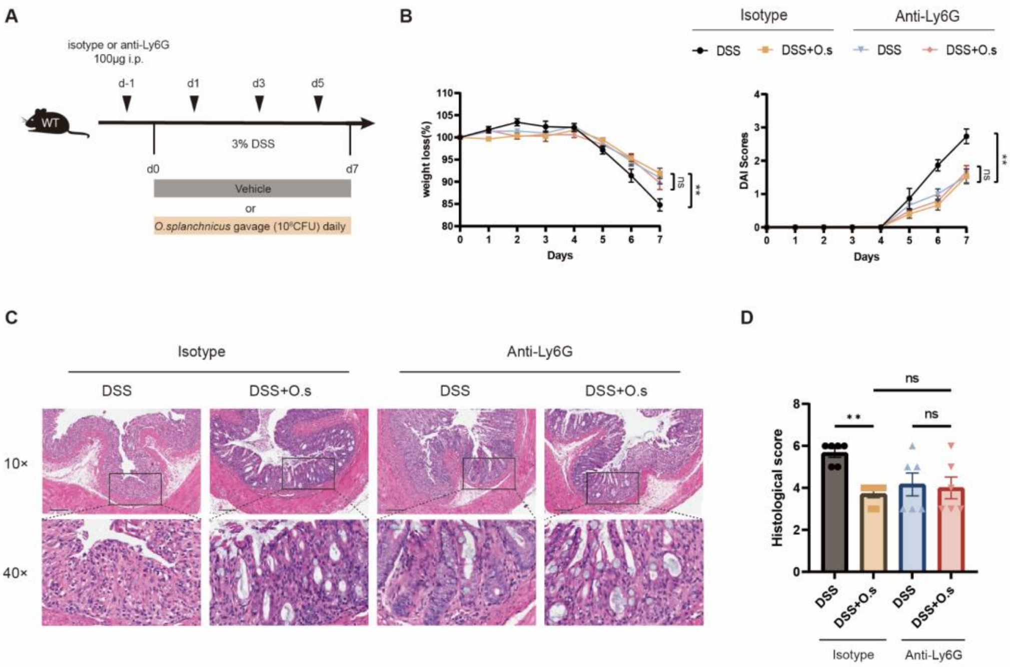

Supplementary Figure 4. (A) Experimental design for the DSS-induced colitis model in wild-type and neutrophils depleted mice by i.p. anti-Ly6G. Mice were administered 3% DSS in drinking water for 7 days, followed by daily oral gavage of *O. splanchnicus* at a concentration of 10^8 CFU/200 μL for 7 days. (B) The weight loss, (C) DAI scores of mice. (C) Representative images of HE staining at 10× and 40× magnification, with comparison of (D) statistical histological scores across groups. Data are shown as mean ± SEM. **, $P < 0.01$, as determined by one-way ANOVA.

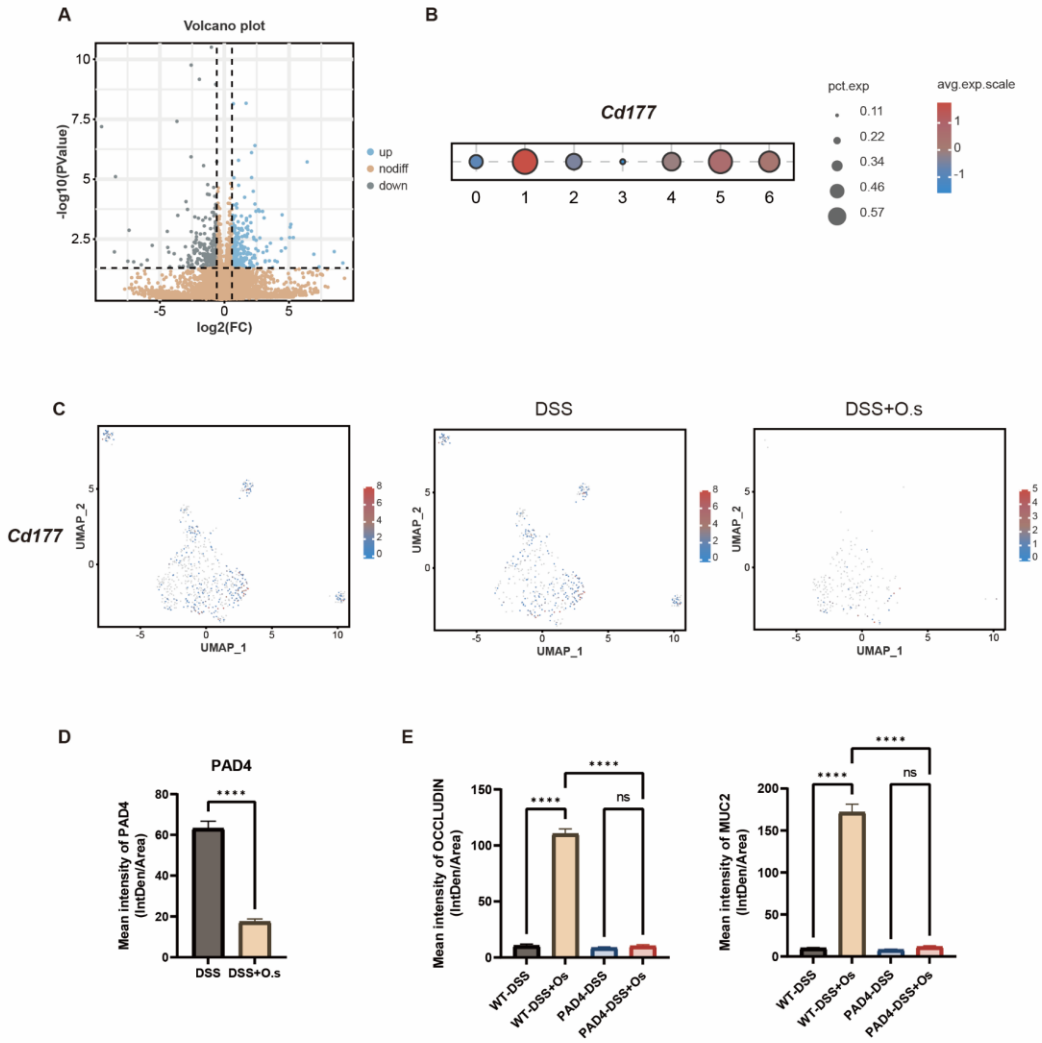

Supplementary Figure 5. (A) The volcano plot shows differentially expressed genes following *O. splanchnicus* intervention based on bulk RNA sequencing analysis. (B) The expression of *Cd177* across clusters of neutrophils, as determined by single cell RNA sequencing. The size of the dots represents the percentage of cells expressing the gene, while the color indicates the level of gene expression. (C) The UMAP plot illustrates *Cd177* expression in colitis mice, with or without *O. splanchnicus* intervention. (D) Statistical analysis of Pad4, (E) OCCLUDIN, and MUC2 intensity in IF images. Data are shown as the mean ± SEM. ****, $P < 0.0001$, as determined by one-way ANOVA.

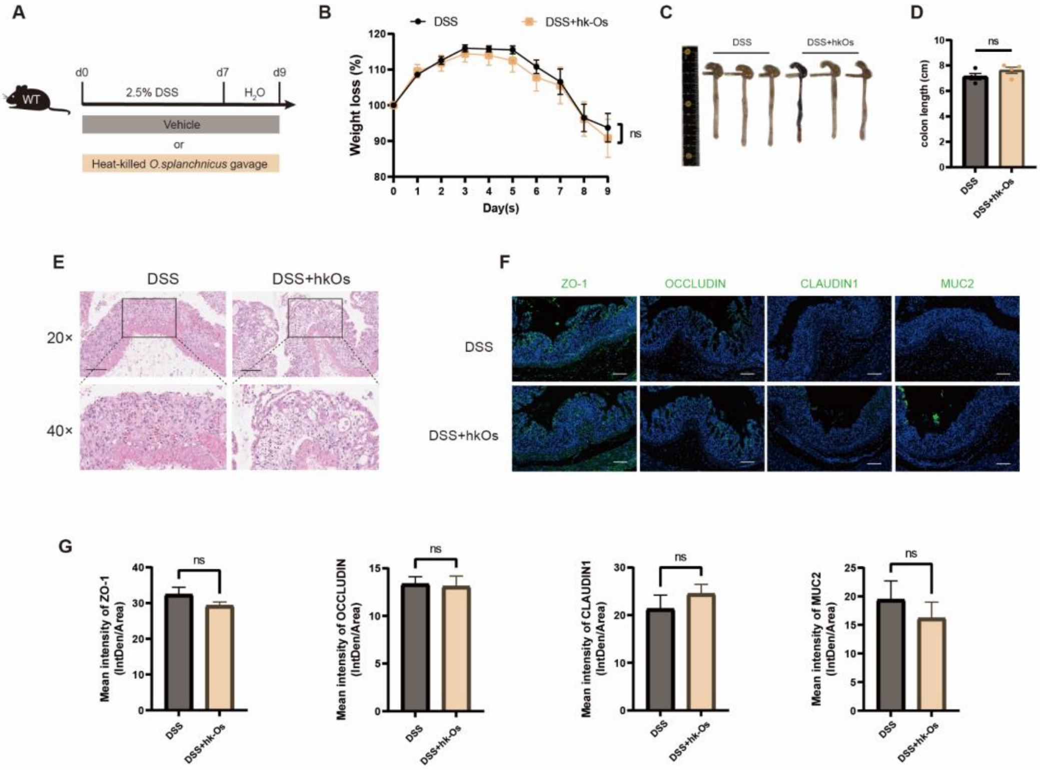

Supplementary Figure 6. (A) Experimental design for 2.5% DSS-induced colitis mice treated with heat-killed *O. splanchnicus.* (B) Weight loss in colitis mice. (C) Representative images of the colon in colitis mice. (D) Statistical analysis of colon length in colitis mice. Data are shown as mean ± SEM; "ns" indicates no significant difference, as determined by an unpaired Student's t-test. (E) Representative images of HE staining at 20× and 40× magnification, along with a comparison of statistical histological scores across groups. (F) Representative images of IF staining for ZO-1 (green), Occludin (green), Claudin1 (green), and Muc2 (green) in colitis mice. Nuclei are stained with DAPI (blue). (G) Statistical analysis of OCCLUDIN, CLAUDIN1, MUC2 intensity in IF images. Data are shown as mean ± SEM; "ns" indicates no significant difference, as determined by an unpaired Student's t-test.

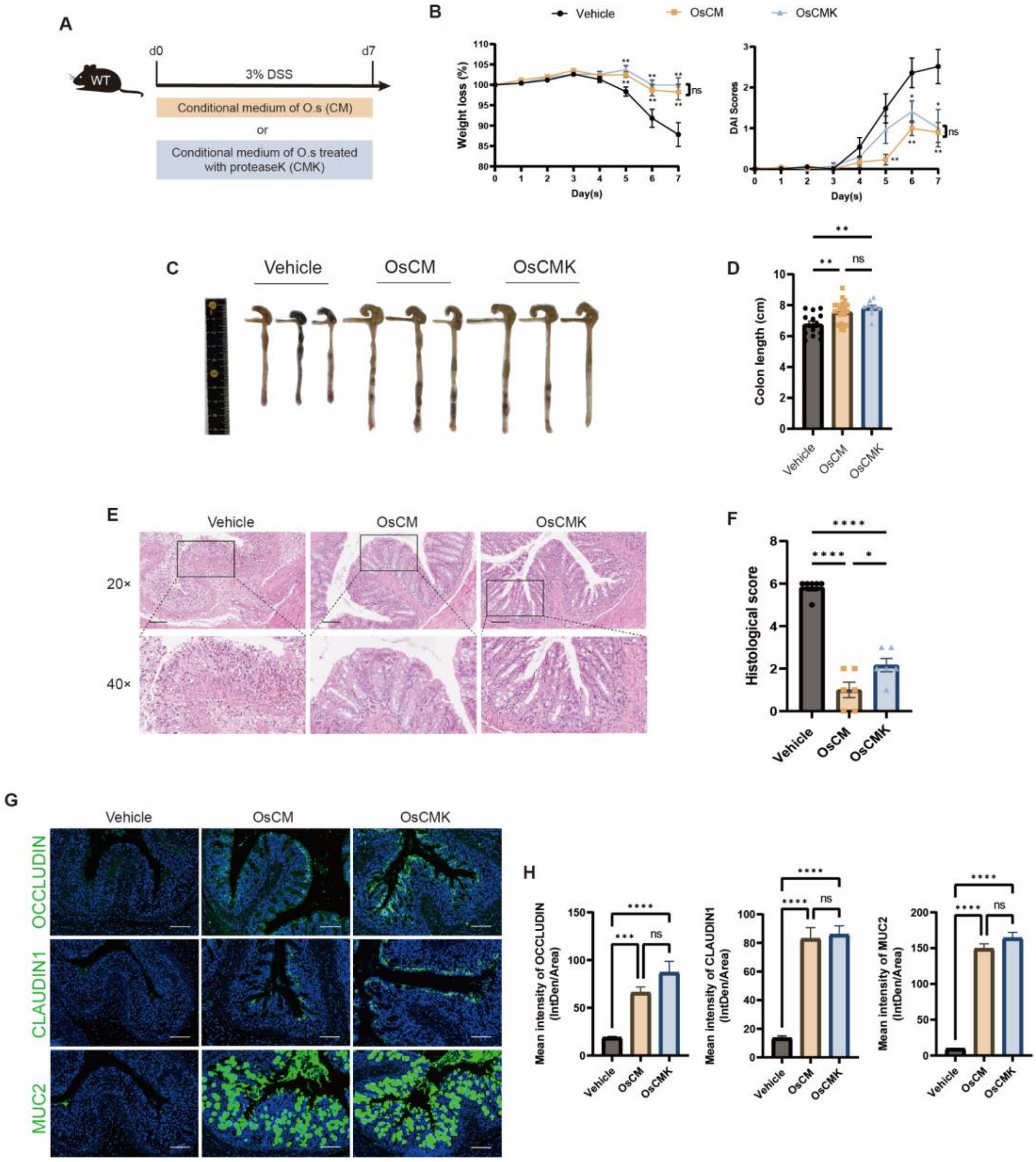

Supplementary Figure 7. (A) Experimental design for 3% DSS-induced colitis mice treated with various fractions of the conditional medium from *O. splanchnicus*. CM, conditional medium obtained after 48 h culturing *O. splanchnicus*; CMK, conditional medium treated with 100 μg/mL protease K, incubated at 50°C for 1 h, and then at 100°C for 5 min. (B) Weight loss and DAI scores. (C) Representative images of the colon. (D) Statistical analysis of colon length across the three groups. Data are presented as mean ± SEM. *, $P < 0.05$; **, $P < 0.01$, as determined by nonparametric Kruskal–Wallis test. (E) Representative images of HE staining at 20× and 40× magnification. (F) Comparison of histological scores across the groups. Data are presented as mean ± SEM. *, $P < 0.05$; ****, $P < 0.0001$, determined by nonparametric Kruskal–Wallis test. (G) Representative images of IF staining for Occludin (green), Claudin1 (green), and Muc2 (green) in colitis mice treated with conditional medium. DAPI was used to stain the nuclei (blue). (H) Statistical analysis of OCCLUDIN, CLAUDIN1, MUC2 intensity in IF images.

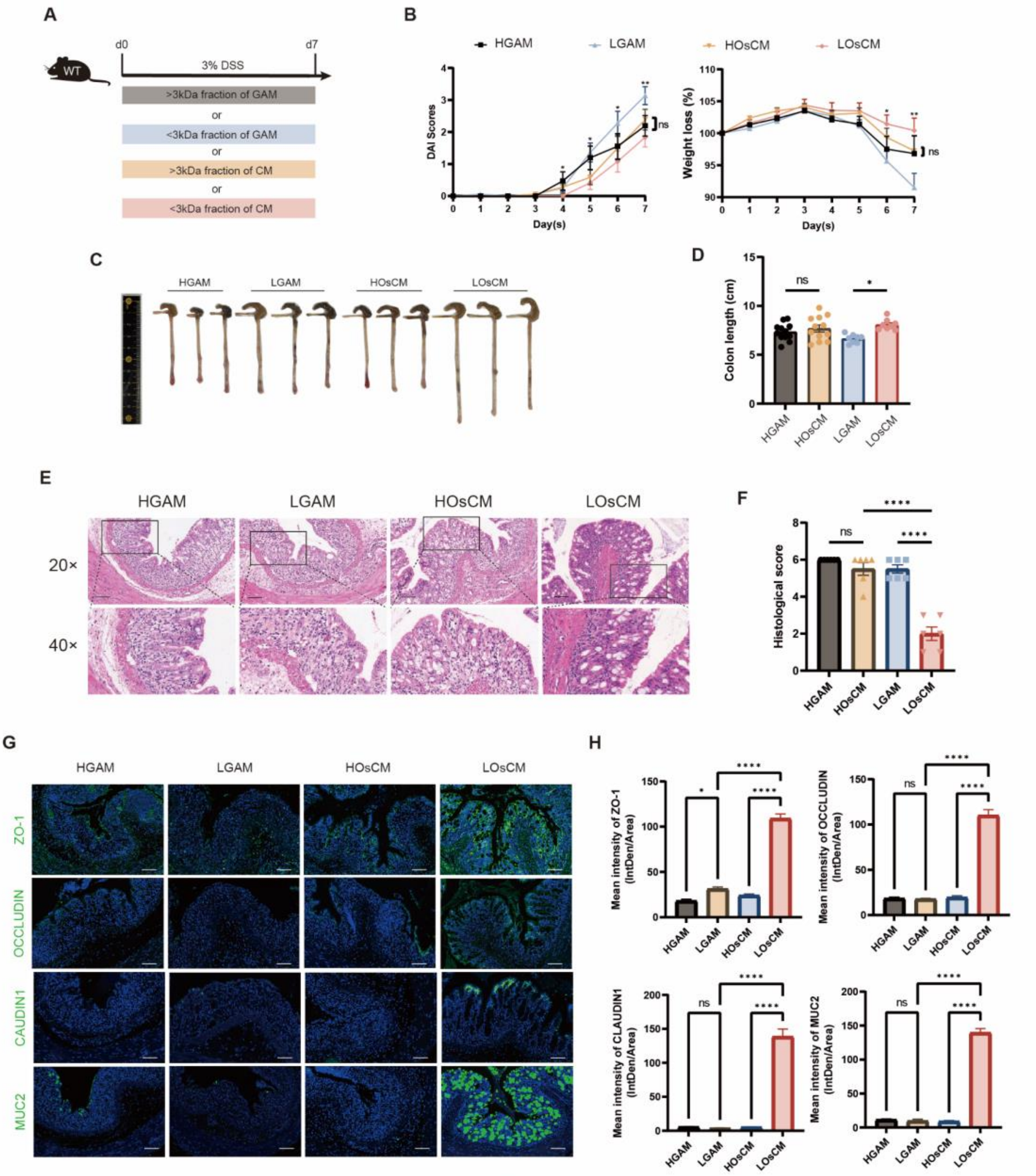

Supplementary Figure 8. (A) Experimental design for 3% DSS-induced colitis mice treated with different fractions of O. splanchnicus conditional medium. Mice were treated with either the >3 kDa or <3 kDa fraction of the conditional medium. GAM served as the vehicle control to assess the effects of conditional medium. (B DAI scores and weight loss. (C) Colon length in the different groups. (D) Representative images of the colon. (E) Representative images of HE staining at 20× and 40× magnification. (F) Statistical comparison of histological scores across the groups.
(G) Representative images of IF staining for Zo-1 (green), Occludin (green), Claudin1 (green), and Muc2 (green) in colitis mice treated with either the >3 kDa or <3 kDa fraction of conditional medium. DAPI was used to stain the nuclei (blue).
(H) Statistical analysis of intensity in IF images. Data are presented as mean ± SEM. *, $P < 0.05$; **, $P < 0.01$; ****, $P < 0.0001$, determined by nonparametric Kruskal–Wallis test.

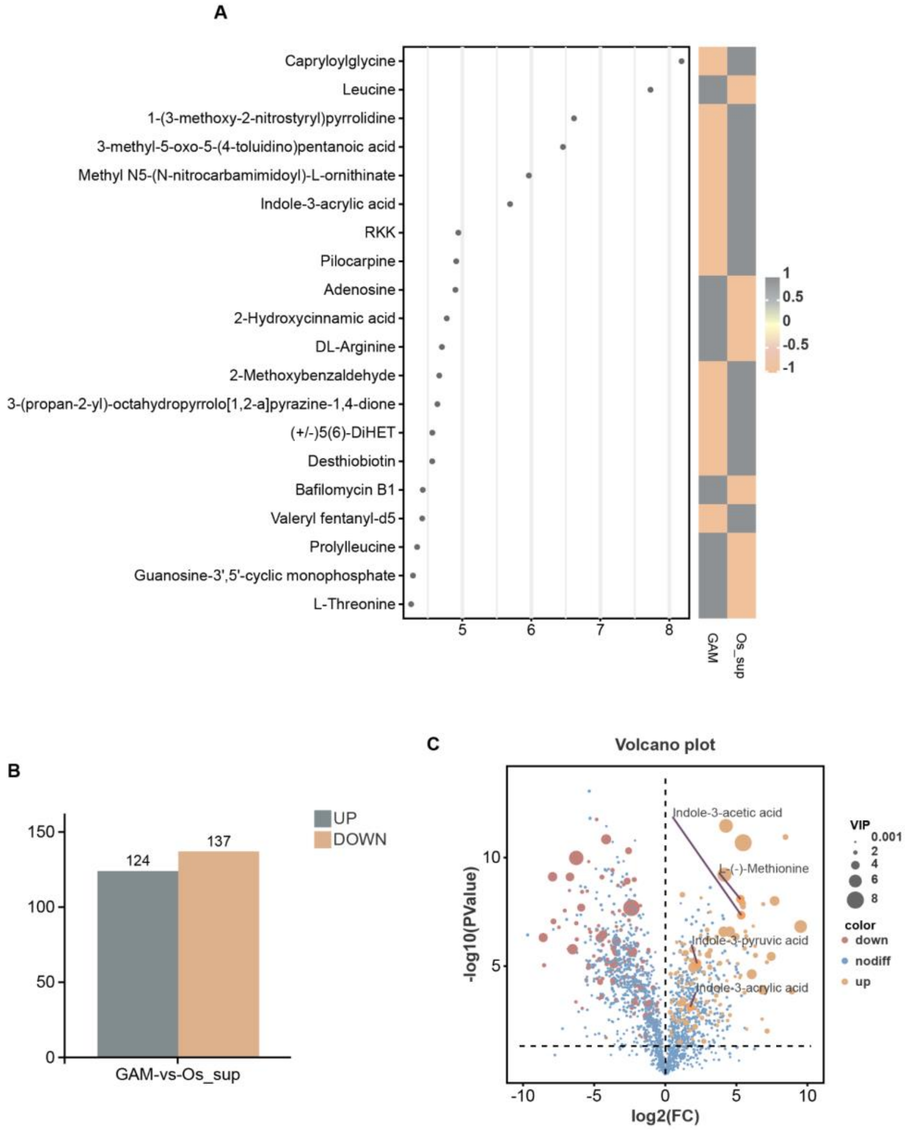

Supplementary Figure 9. (A) The VIP scores of metabolites in two groups. (B) The up and down-regulated differential metabolites in the conditioned medium of *O.splanchnicus*. (C) The Volcano plot image of metabolites.

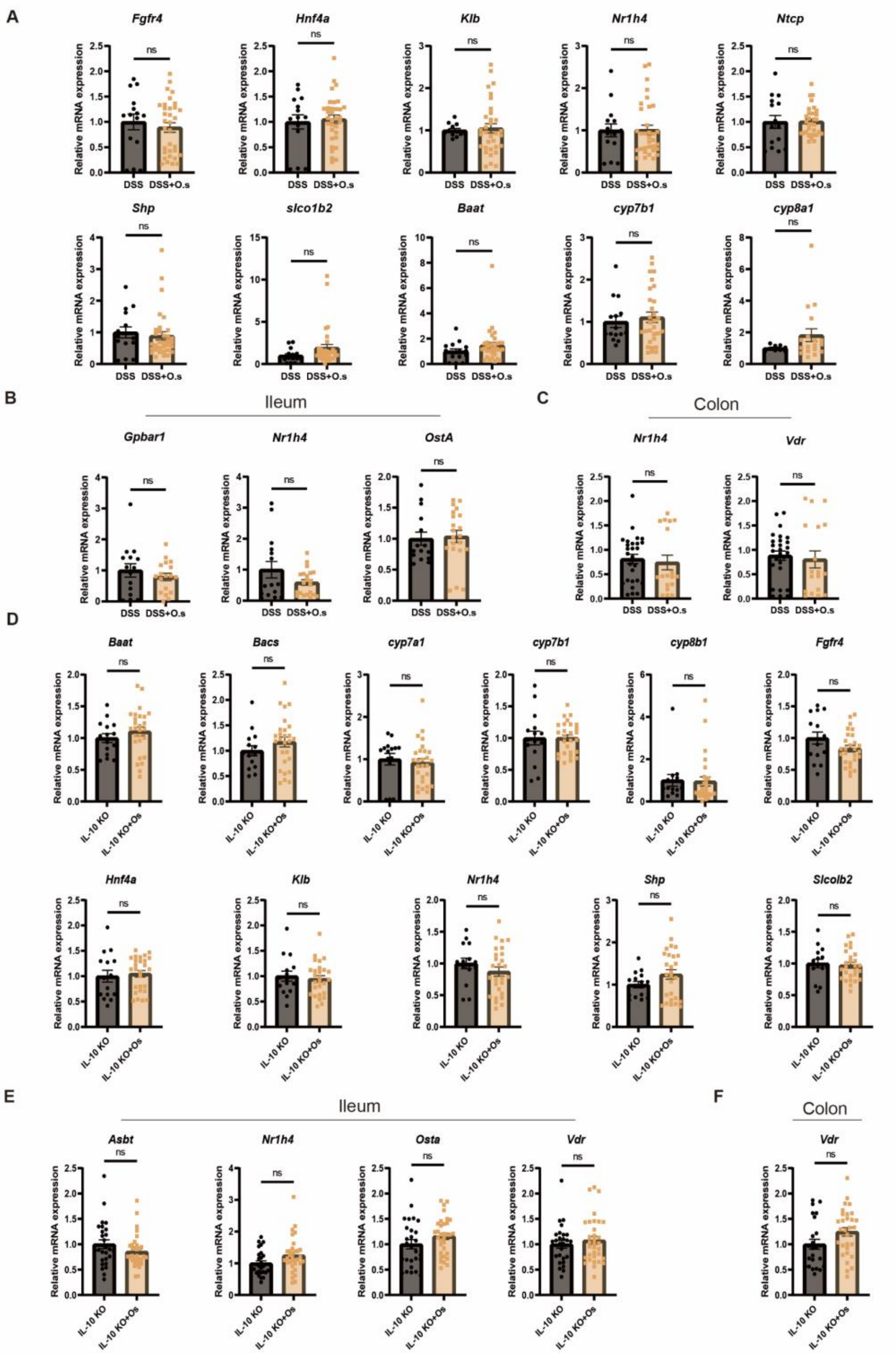

Supplementary Figure 10. (A) mRNA expression of bile acid synthesis-related genes in the liver of DSS-induced colitis mice and (D) *Il-10*$^{-/-}$ mice. (B) mRNA expression of bile acid transporters in the ileum of DSS-induced colitis mice and (E) *Il-10*$^{-/-}$ mice. (C) mRNA expression of bile acid receptors in the colon of DSS-induced colitis mice and (F) *Il-10*$^{-/-}$ mice. Data are presented as mean ± SEM. "ns" denotes no significant difference, as determined by an unpaired Student's t-test.

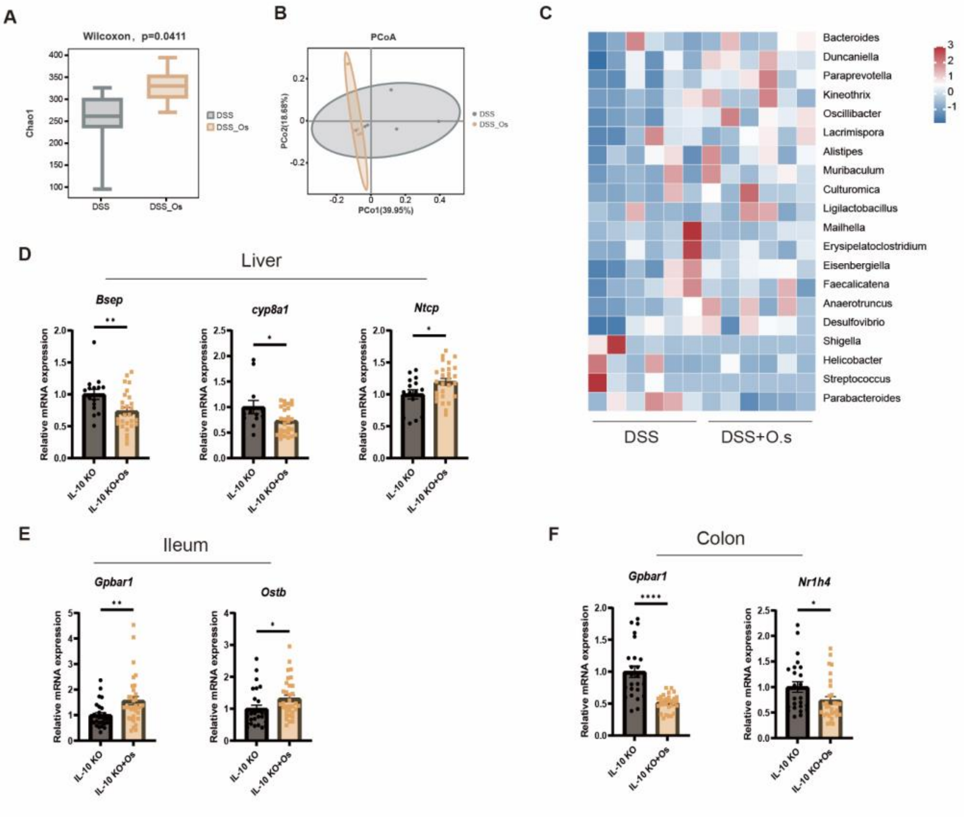

Supplementary Figure 11. (A) α-diversity as measured by the Chao1 index in the gut microbiota of colitis mice after *O. splanchnicus* intervention, assessed by 16S rRNA sequencing. Statistical analysis was performed using the Wilcoxon test. (B) PCoA depicting the β-diversity of the gut microbiota following *O. splanchnicus* intervention. (C) Heatmap showing the top 20 differential taxa at the genus level in colitis mice treated with *O. splanchnicus*. (D) The mRNA expression of bile acid synthesis-related genes *Bsep*, *Cyp8a1*, and *Ntcp* in the liver of *Il-10*$^{-/-}$ mice after *O. splanchnicus* intervention. (E) The mRNA expression of bile acid transporters *Gpbar1* and *Ostb* in the ileum of *Il-10*$^{-/-}$ mice after *O. splanchnicus* intervention. (F) The mRNA expression of bile acid receptors *Gpbar1* and *Nr1h4* in the colon of *Il-10*$^{-/-}$ mice after *O. splanchnicus* intervention. Data are presented as mean ± SEM. *, $P < 0.05$; **, $P < 0.01$; ****, $P < 0.0001$, as determined by unpaired Student's t test.

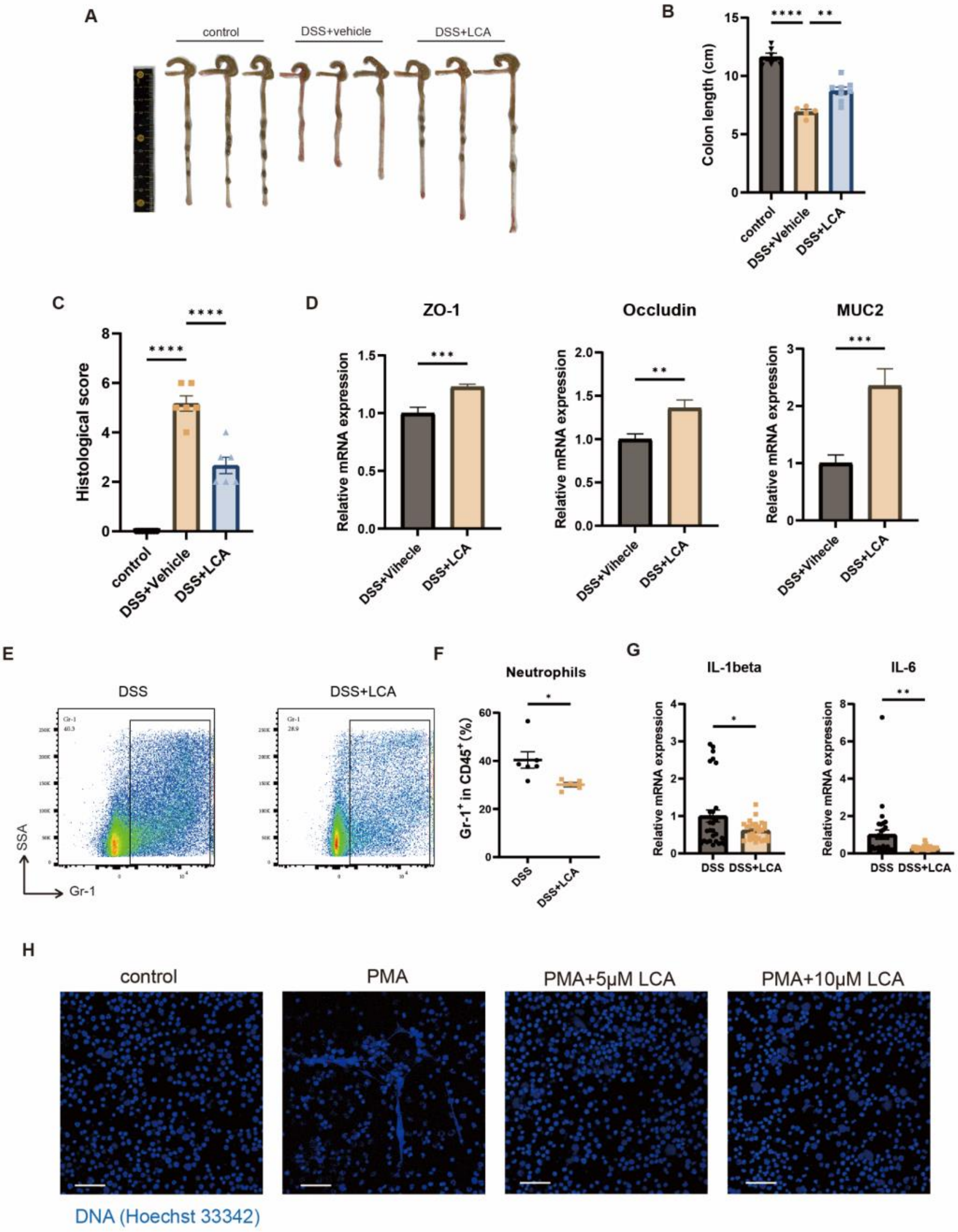

Supplementary Figure 12. (A) Representative images of the colon, (B) colon length in the three groups. (C) Statistical analysis of histological scores.Data are shown as mean ± SEM. *, $P < 0.05$, **, $P < 0.01$, as determined by one-way ANOVA. (D) The relative mRNA expression of Zo-1, Occludin, Muc2 in colon. (E) Representative images of Gr-1$^+$ neutrophils by flow cytometry. (F) Statistical analysis of Gr-1$^+$ neutrophils. (G) The relative mRNA expression of Il-1β and Il-6 in colon. Data are shown as mean ± SEM. *, $P < 0.05$, **, $P < 0.01$, as determined by an unpaired Student's t-test. (H) Representative images of cells stained with Hoechst 33342.

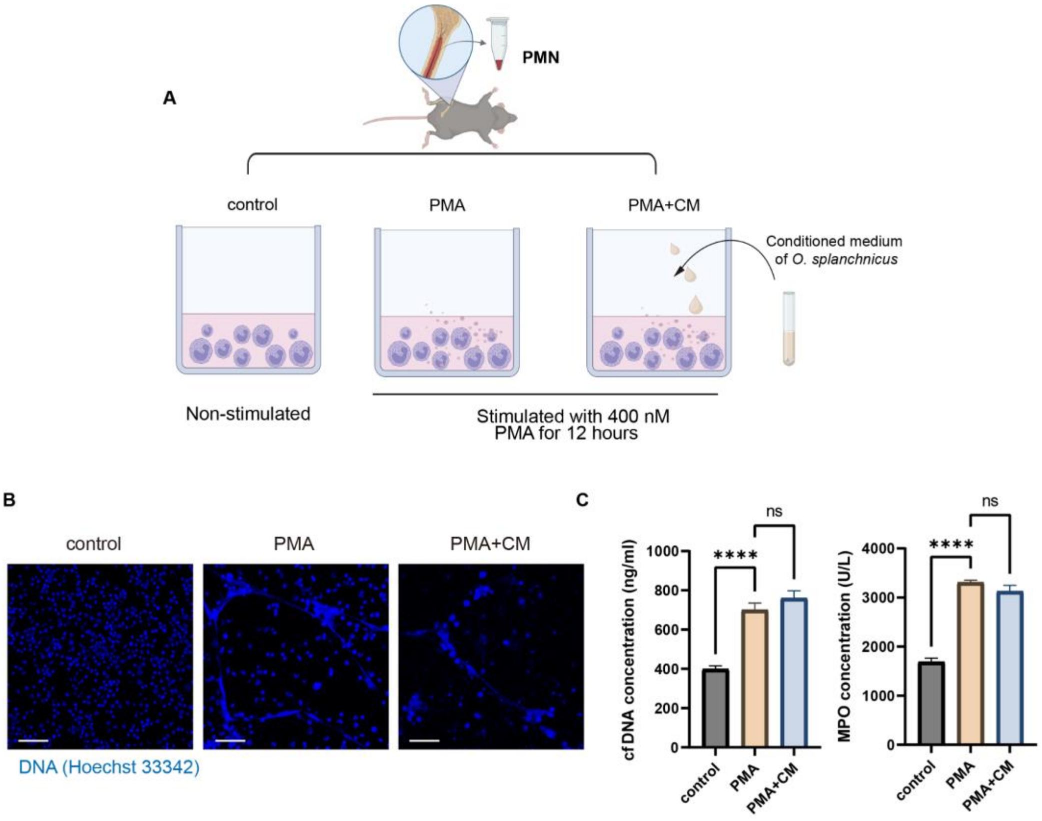

Supplementary Figure 13. (A) Experimental design for coculture isolated murine neutrophils with conditioned medium of *O.splanchnicus*. (B) Representative images of cells stained with Hoechst 33342 in each group. (C) The concentration of cfDNA and MPO from the supernatant of cell after coculture. Data are presented as mean ± SEM (n = 6). ****, *P* < 0.0001; as determined by nonparametric Kruskal–Wallis test.

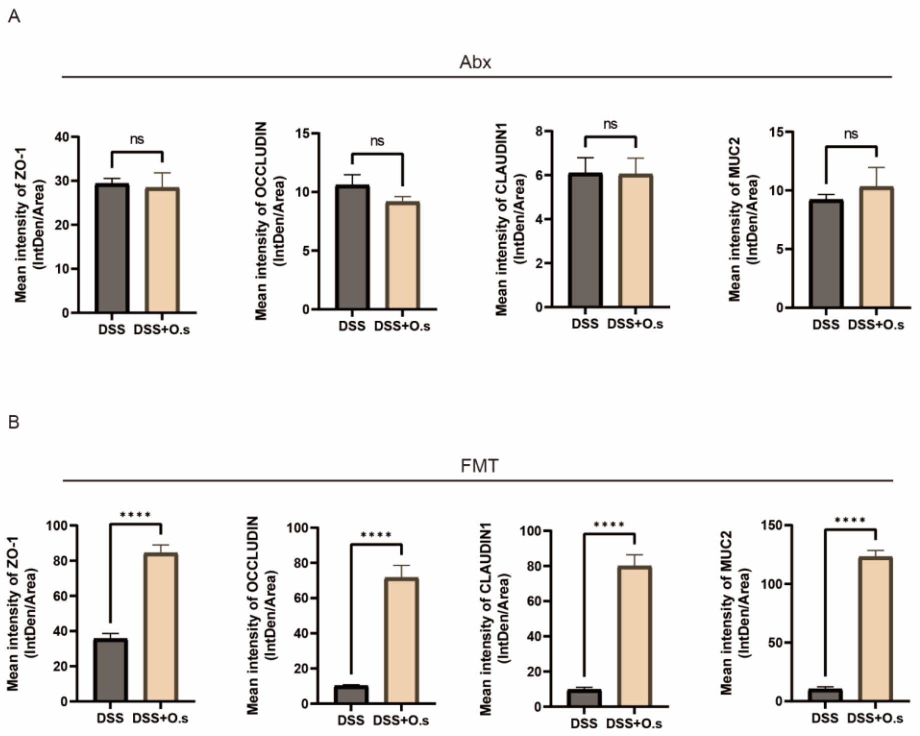

Supplementary Figure 14. (A) Statistical analysis of ZO-1, OOCLUDIN, CLAUDIN1, and MUC2 intensity in IF images in the DSS and DSS+O.s group after antibiotic cocktails pretreatment. (B) Statistical analysis of ZO-1, OOCLUDIN, CLAUDIN1, and MUC2 intensity in IF images in the DSS and DSS+O.s group after FMT intervention. Data are shown as mean ± SEM. ****, *P* < 0.0001, as determined by an unpaired Student's t-test.

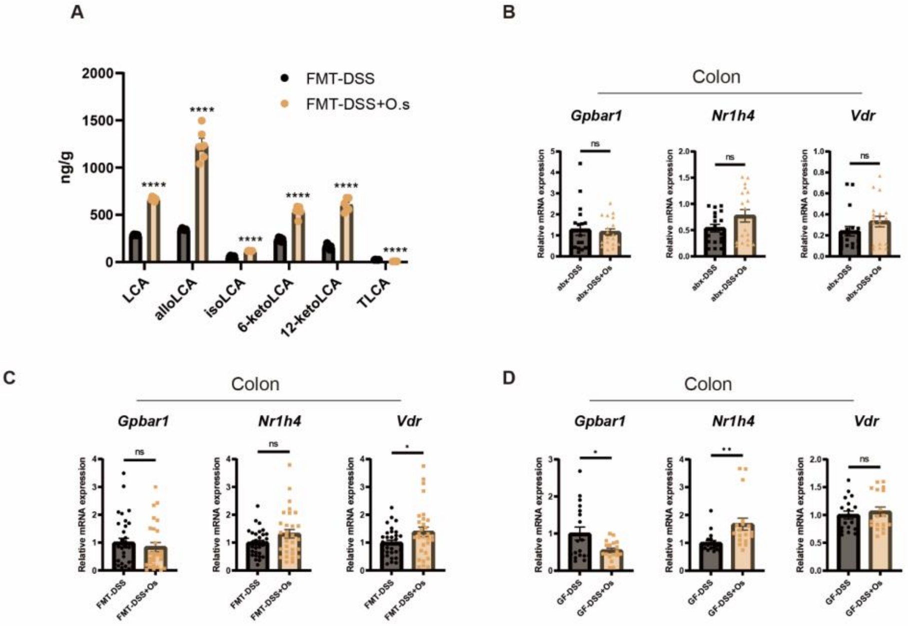

Supplementary Figure 15. (A) Concentration of LCA and its derivatives in the feces of colitis mice that received FMT treatment. Data are shown as mean ± SEM. ****, $P < 0.0001$, as determined by an unpaired Student's t-test. (B) mRNA expression levels of bile acid receptors *Gpbar1*, *Nr1h4*, and *Vdr* in the colon of colitis mice with antibiotic pretreatment. (C) mRNA expression levels of bile acid receptors *Gpbar1*, *Nr1h4*, and *Vdr* in the colon of colitis mice with FMT treatment. (D) mRNA expression levels of bile acid receptors *Gpbar1*, *Nr1h4*, and *Vdr* in the colon of germ-free mice. Data are shown as mean ± SEM. *, $P < 0.05$; **, $P < 0.01$, as determined by an unpaired Student's t-test.

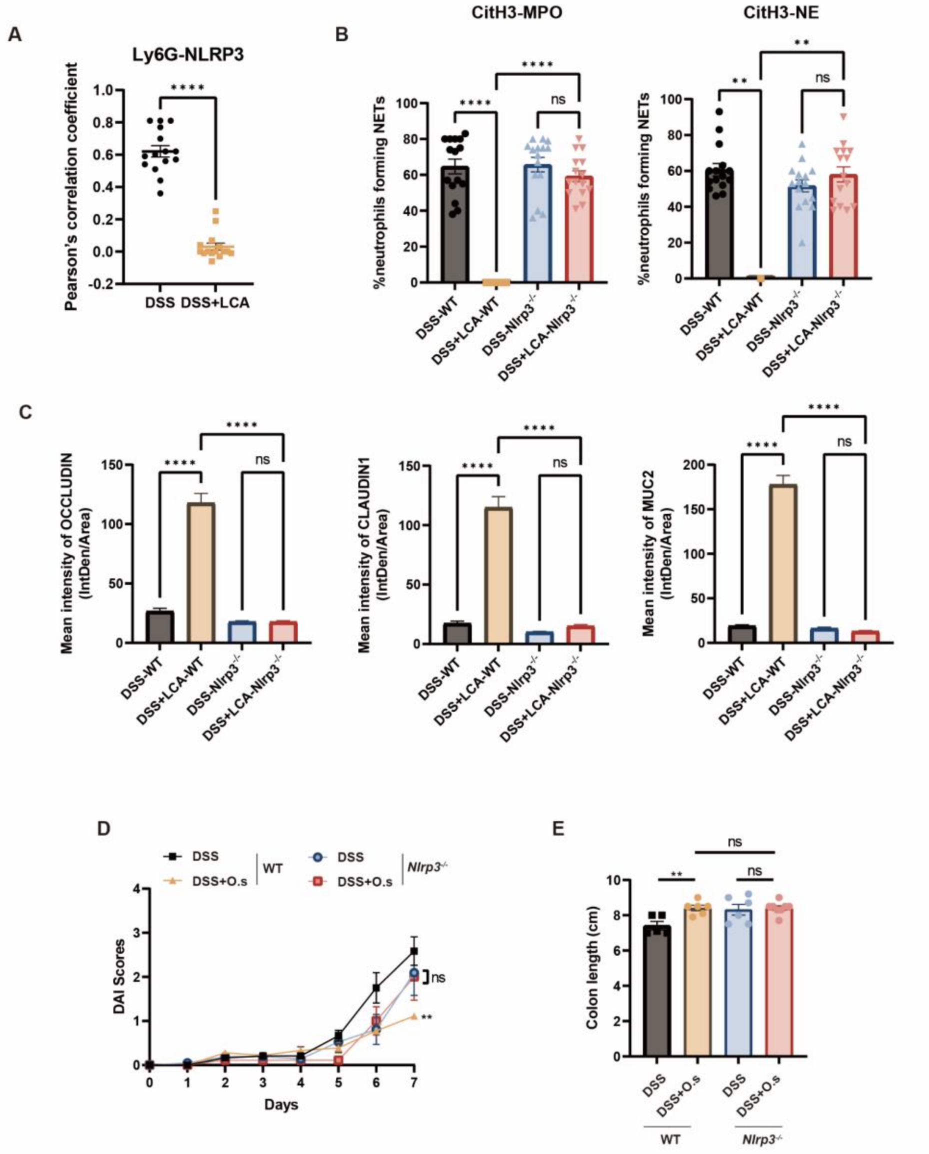

Supplementary Figure 16. (A) The colocolization analysis of Ly6G and Nlrp3 by Pearson's correlation coefficient in IF images. (B) The percentage of NETs forming neutrophils in groups. (C) Statistical analysis OCCLUDIN, CLAUDIN1, and MUC2 intensity in IF images. (D) DAI scores of wild-type mice and *Nlrp3*$^{-/-}$ mice following *O. splanchnicus* intervention. Colitis was induced in the mice with 3% DSS for 7 days. (E) Statistical analysis of colon length in all groups. Data are shown as mean ± SEM. **, $P < 0.01$, as determined by the nonparametric Kruskal–Wallis test.

Table S1 The sequence of primer.

Gene	Sequence
Zo-1	*Forward: 5'- CAAAGCCCACCAAGGTCAC-3'*
	Reverse: 5'- TCTCTTTCCGAGGCATTAGCA-3'
Muc2	*Forward: 5'-CCCAGAAGGGACTGTGTATG-3'*
	Reverse: 5'- TGCAGACACACTGCTCACA-3'
Occludin	*Forward: 5'- TGGCAAGCGATCATACCCAGAG-3'*
	Reverse: 5'- CTGCCTGAAGTCATCCACACTC-3'
O.splanchnicus	*Forward: 5'- ATGTAATGATGAGCACTCTAACGG-3'*
	Reverse: 5'- GGCTTTTGAGATTGGCATCC-3'
UNI16S	*Forward: 5'- GGTGAATACGTTCCCGG-3'*
	Reverse: 5'- TACGGCTACCTTGTTACGACTT-3'
Il-6	*Forward: 5'-GTTGCCTTCTTGGGACTGATG-3'*
	Reverse: 5'- ATTGCCATTGCACAACTCTTT-3'
Il-1β	*Forward: 5'-AGAGCATCCAGCTTCAAATCTC-3'*
	Reverse: 5'- CAGTTGTCTAATGGGAACGTCA-3'
Bacs	*Forward: 5'- CTGCGGTACTTGTGTAACGTCC-3'*
	Reverse: 5'- TCCGAATGGGACCAAAGCGTTG-3'
Bsep	*Forward: 5'- CCTTGGTAGAGAAGAGGCGACA-3'*
	Reverse: 5'- ATGGCTACCCTTTGCTTCTGCC-3'
Cyp2c70	*Forward: 5'- TGGGCTTTTGCTCCTGCTGAAG-3'*
	Reverse: 5'- TCAGTGTACGGCATGTGGTTCC-3'
Cyp7a1	*Forward: 5'- CACCATTCCTGCAACCTTCTGG-3'*
	Reverse: 5'- ATGGCATTCCCTCCAGAGCTGA-3'
Cyp8b1	*Forward: 5'- CATGAAGGCTGTGCGTGAGGAA-3'*
	Reverse: 5'- CATCACGCTGTCCAACACTGGA-3'
Cyp27a1	*Forward: 5'- TCAGGAGACCATCGGCACCTTT-3'*
	Reverse: 5'- CCAGTCACTTCCTTGTGCAAGG-3'
Ostb	*Forward: 5'- CAAGCATGTTCCTCCTGAGAAGG-3'*
	Reverse: 5'- CTCTTAGGAAGACCTGGCTGTTG-3'

Vdr	*Forward: 5'- GCTCAAACGCTGCGTGGACATT-3'*
	Reverse: 5'- GGATGGCGATAATGTGCTGTTGC-3'
Astb	*Forward: 5'- GGTTTCTTCCTGGCTAGACTAGC-3'*
	Reverse: 5'- GGAAGGTGAACACCAGGTTGAG-3'
Gpbar1	*Forward: 5'- CACTGCTCTTCTTGCTGTGTTGG-3'*
	Reverse: 5'- GAGCGATAACAGAGTTCCAGGC-3'
Fgfr4	*Forward: 5'- TCCGACAAGGATTTGGCAGACC-3'*
	Reverse: 5'- TGGCGGCACATTCCACAATCAC-3'
Hnf4a	*Forward: 5'- TGCGAACTCCTTCTGGATGACC-3'*
	Reverse: 5'- CAGCACGTCCTTAAACACCATGG-3'
Klb	*Forward: 5'- GAAAGAGTCCACGCCAGACATG-3'*
	Reverse: 5'- CAGGTGAGGATCGGTAAACTGC-3'
Nr1h4	*Forward: 5'- GGGATGAGTGTGAAGCCAGCTA-3'*
	Reverse: 5'- GTGGCTGAACTTGAGGAAACGG-3'
Ntcp	*Forward: 5'- CCTGATGCCTTTCACTGGCTTC-3'*
	Reverse: 5'- GGATGGTAGAACAGAGTTGGACG-3'
Shp	*Forward: 5'- CCAAGGAGTATGCGTACCTGAAG-3'*
	Reverse: 5'- GCTCCAAGACTTCACACAGTGC-3'
Slco1b2	*Forward: 5'- GCAATGATCGGACCAATCCTTGG-3'*
	Reverse: 5'- CCAACGAGCATCCTGAGGAGTT-3'
Baat	*Forward: 5'- GGATAGCCTGACTCTGGAAAGG-3'*
	Reverse: 5'- CAATCCACCAGCACCTCCAAAC-3'
Cyp7b1	*Forward: 5'- CGGAAATCTTCGATGCTCCAAAG-3'*
	Reverse: 5'- GCTTGTTCCGAGTCCAAAAGGC-3'
Cyp8a1	*Forward: 5'- GGAGACAGGTCTCCTTGAGTTC-3'*
	Reverse: 5'- AACATCCGCTGAGTGGACACGA-3'
Osta	*Forward: 5'- GCCTGCCATTTTCTCCATCTTGG-3'*
	Reverse: 5'- CAGCACTGTCATCAGGAAGGTC-3'
Actb	*Forward: 5'- CATTGCTGACAGGATGCAGAAGG-3'*

Forward: 5'- TGCTGGAAGGTGGACAGTGAGG-3'
